# Elucidating heterogeneous photocatalytic superiority of microporous porphyrin organic cage

**DOI:** 10.1038/s41467-020-14831-x

**Published:** 2020-02-26

**Authors:** Chao Liu, Kunhui Liu, Chiming Wang, Heyuan Liu, Hailong Wang, Hongmei Su, Xiyou Li, Banglin Chen, Jianzhuang Jiang

**Affiliations:** 1 Department of Chemistry, Beijing Key Laboratory for Science and Application of Functional Molecular and Crystalline Materials, University of Science and Technology Beijing, Beijing, 100083 China; 2 College of Chemistry, Beijing Normal University, Beijing, 100875 China; 3 School of Materials Science and Engineering, China University of Petroleum (East China), Qingdao, 266580 China; 4 Department of Chemistry, University of Texas at San Antonio, San Antonio, TX 78249-0698 USA

**Keywords:** Organocatalysis, Photocatalysis, Metal-organic frameworks

## Abstract

The investigation on the catalytic properties of porous organic cages is still in an initial stage. Herein, the reaction of cyclohexanediamine with 5,15-di[3’,5’-diformyl(1,1’-biphenyl)]porphyrin affords a porphyrin tubular organic cage, PTC-1(2H). Transient absorption spectroscopy in solution reveals much prolonged triplet lifetime of PTC-1(2H) relative to monomer reference, illustrating the unique photophysical behavior of cagelike photosensitizer. The long triplet lifetime ensures high-efficiency singlet oxygen evolution according to homogeneous photo-bleach experiment, electron spin-resonance spectroscopy, and aerobic photo-oxidation of benzylamine. Furthermore, microporous supramolecular framework of PTC-1(2H) is able to promote the heterogeneous photo-oxidation of various primary amines with conversion efficiency above 99% under visible light irradiation. These results indicate the great application potentials of porous organic cages in heterogeneous phase.

## Abstract (web summary)

The investigation on the catalytic properties of porous organic cages is still in an initial stage. Herein, the heterogeneous photocatalytic superiority of microporous porphyrin organic cage has been clearly elucidated due to unique cage-induced long triplet lifetime and porous structure.

## Introduction

Visible light photocatalysis provides a green and sustainable approach for the synthesis of valuable organic chemicals by conceptually harnessing solar energy^1–6^, though it is still a great challenge to directly convert visible light to chemical energy because of the lack of absorption in this visible light region for most organic compounds. It has been established that the excited triplet states of some visible light-absorbing molecules are able to initiate the catalytic cycle via photo-induced electron/energy transfer process^1–6^. By virtue of the strong visible light absorption, long triplet lifetime, and high triplet quantum yield, porphyrin derivatives have been extensively utilized as one of the most powerful photocatalysts toward aerobic organic reactions^7–18^. Their practical applications have been retarded by aggregation-induced deactivation of photosensitized performance and low-density solar energy^5,15^; it is therefore of great importance to develop excellent molecular porphyrin photocatalysts with improved absorption and excited states for facilitating photon conversion.

Molecular cages such as metal–organic coordination cages and porous organic cages are fabricated from discrete modules mainly depending on coordination and covalent bonds, respectively^19–49^. These unique synthetic hosts with well-defined cavities are capable of offering diverse weak interactions to recognize and encapsulate organic substrates^46^, constrain their orientations^47^, stabilize intermediates^48^, and mediate reaction kinetics, and thus used for their diverse applications^49^. Although the enzyme-like catalytic activities have been revealed for such special hosts by grafting/post-trapping catalytically active sites on cage skeletons or in cavities, respectively^25,44,45,50–52^, visible light photocatalytic molecular cages toward organic reactions have been still very rarely realized because of the low quantum yield of excited states attributed to the cage-induced self-quenching effect^23,53–55^. It has been envisioned that the ultrafast photo-induced energy/electron communication between hosts and cavity-confined guests in a close distance^23^ may facilitate the rapid conversion of reaction reagents. In addition, the supramolecular frameworks of molecular cages will possess not only the intrinsic intracage cavities but also the intercage porosities^19,27,34^, providing a renewable platform for heterogenizing homogeneous photocatalysts. With these considerations in our mind, we plan to develop visible light photocatalytic molecular cages constructed from porphyrin building units for their photo-induced heterogeneous catalysis.

In the present work, a [3+6] porous tubular organic cage consisting of three porphyrin units, PTC-1(2H), has been constructed and characterized by single crystal X-ray diffraction analysis. Transient absorption spectroscopy in solution reveals its prolonged triplet lifetime relative to porphyrin monomer, ensuring this kind of porphyrin molecular cage to efficiently promote the visible light-driven evolution of reactive oxygen species (ROS) and thus the aerobic photo-oxidation of benzylamine in homogeneous phase. Furthermore, such prominent molecular photocatalytic activity, in combination with the porous structure of the cage-based supramolecular framework, renders the solid PTC-1(2H) to exhibit 2–5 times faster reaction rate in heterogeneous visible light catalysis of the aerobic photo-oxidation of primary amines than the reference monomer solid and two well-known porphyrin-containing metal‒organic frameworks (MOFs), PCN-222 and PCN-224. These results disclose the great application potential of porous organic cages in visible light heterogeneous photocatalysis.

## Results

### Synthesis of porphyrin organic cage PTC-1(2H)

**Fig. 1:**
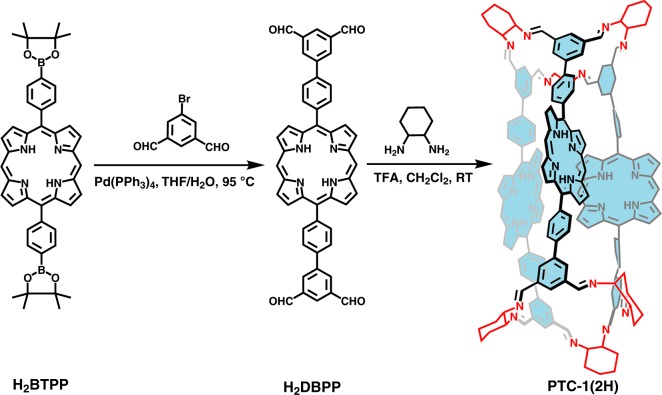
Synthesis of tubular porphyrin organic cage. The synthetic route of PTC-1(2H) includes Suzuki–Miyaura cross-coupling between 5-bromoisophthalaldehyde and 5,15-bis[4-(4,4,5,5-tetramethyl-1,3,2-dioxaborolan-2-yl)phenyl]porphyrin (H_2_BTPP) together with imine condensation reaction of enantiomerically pure cyclohexanediamine with 5,15-di[3’,5’-diformyl-(1,1’-biphenyl)]porphyrin (H_2_DBPP).

Inspired by the pioneering work of Cooper and co-workers on fabricating the [3+6] porous organic cages including TCC1, TCC2, and TCC3^56^, a tubular organic cage, PTC-1(2H), composed of porphyrin units was designed through the synthesis pathway (Fig. 1). To direct the synthesis, theoretical simulation over the reaction between metal-free 5,15-di[3’,5’-diformyl-(1,1’-biphenyl)]porphyrin (H_2_DBPP) and cyclohexanediamine was carried out, giving a formation energy of −79 kcal mol^−1^ for the metal-free porphyrin organic cage (Supplementary Data 1), consolidating the synthetic feasibility of PTC-1(2H) from thermodynamics by means of such a reaction route; for details, please see Supplementary Information. Subsequent reaction of H_2_DBPP with enantiomerically pure cyclohexanediamine afforded two enantiomers of PTC-1(2H) in a good yield of >85% with the help of trifluoroacetic acid as catalyst. This cage and a monomeric metal-free 5,15-di[3’,5’-cyclohexyliminomethyl-1,1’-biphenyl]porphyrin (H_2_CBPP) were characterized by a series of spectroscopic methods, including nuclear magnetic resonance (NMR), ultraviolet–visible, circular dichroism, and mass spectroscopy (Table 1 and Supplementary Figs. 1–12 and Supplementary Table 1).

**Table 1:** Photophysical and electrochemical data for H_2_CBPP and PTC-1(2H).

Compound	UV/vis spectra in toluene	fluorescence spectra in toluene			
	*λ*_max_ (nm)	*λ*_ex_ (nm)	*λ*_em_ (nm)	*τ* (ns)	*φ* (%)
PTC-1(2H)	397, 506, 542, 580, 635	405	638, 702	9.80	1.82
H_2_CBPP	412, 504, 540, 579, 635	405	638, 700	9.87	1.86

Compound	CV spectra^a^ (vs. SCE)		TA spectra in toluene		
	Red_1_ (V)	Oxd_1_ (V)	TA band (nm) in N_2_	*τ* (μs)^b^	*τ* (μs)^c^
PTC-1(2H)	−0.97	0.98	430–500	102.20	0.33
H_2_CBPP	−0.98	1.10	425–500	53.98	0.25

^a^In CH_2_Cl_2_.

^b^In N_2_.

^c^In air.

### Molecular and supramolecular structures of PTC-1(2H)

**Fig. 2:**
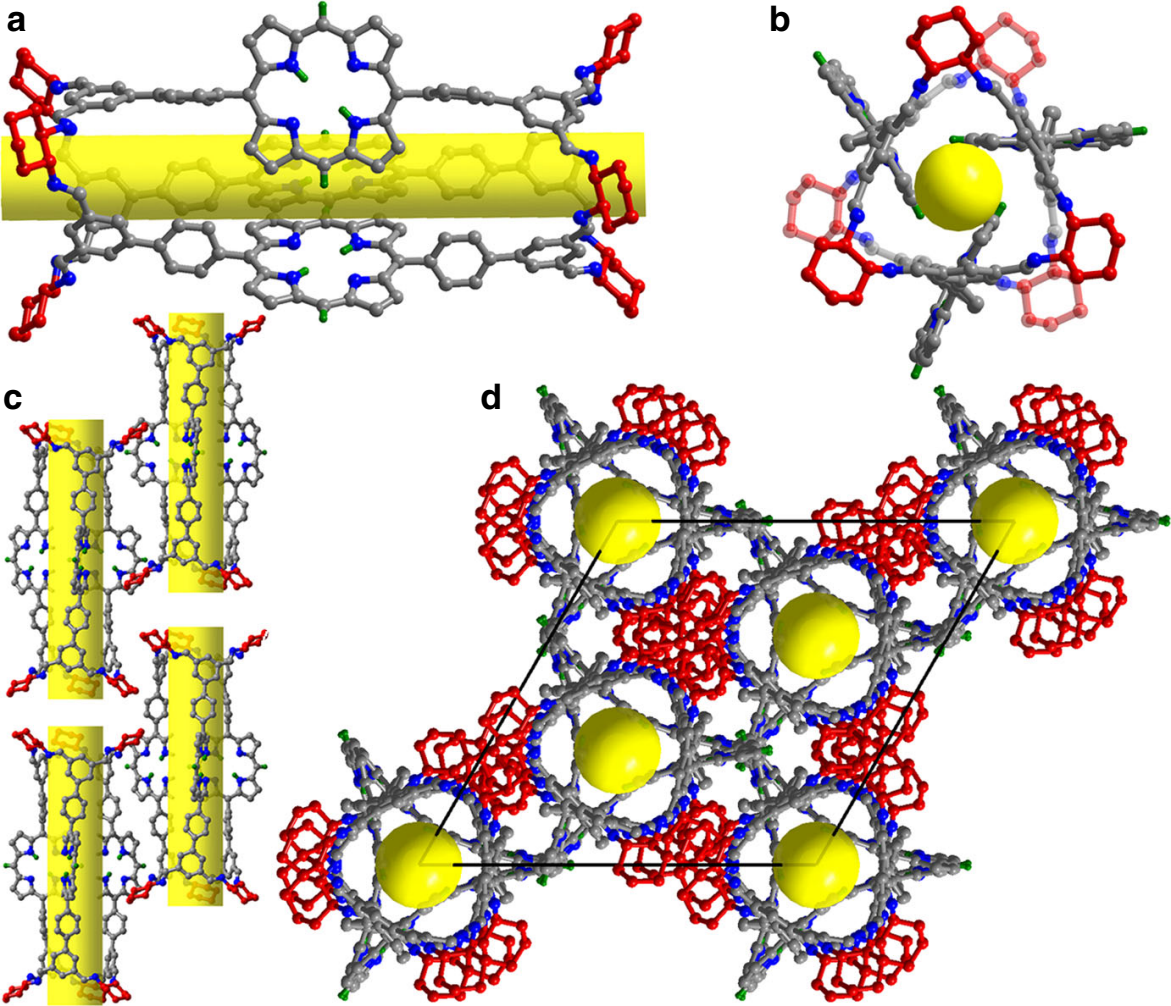
Single crystal structure of molecular organic cage (R)-PTC-1(2H). **a** Side view and **b** top view; **c** window-to-window stacking mode of neighboring molecular organic cages; **d** packing profile along the direction of [001] (porphyrin C, gray; cyclohexanediamine C, red; N, blue; H, green; yellow tubes and balls represent the open one-dimensional channel and window, respectively; all selected hydrogen atoms omitted for clarity).

Purple cubic single crystals of this pair of enantiomers suitable for X-ray diffraction analysis were obtained by diffusing methanol into the chloroform solution of the corresponding compound. The crystal structures of both enantiomers, (*R*)-PTC-1(2H) (Fig. 2) and (*S*)-PTC-1(2H) (Supplementary Fig. 13), were determined by X-ray diffraction analysis. Both isostructural enantiomers crystallize in the trigonal system with a chiral *R*32 space group (Supplementary Table 2), showing six cage molecules per unit cell. Figures 2a and 2b display the molecular structure of (*R*)-PTC-1(2H) as representative in side view and top view, respectively. Each cage molecule is composed of 3 porphyrin segments and 6 cyclohexanediimine groups linked by 12 imine bonds, forming a tubular structure with the length of 3.3 nm, which appears to represent one of the longest molecular tube^19–55^. For each cage molecule, the separation of *meso*-positional H atom of each porphyrin unit to the N4 mean plane of neighboring porphyrin unit amounts to 2.8510(8) Å, indicating the presence of intramolecular C‒H–π interaction. As shown in the packing diagram for (*R*)-PTC-1(2H) (Fig. 2c, d), the neighboring molecular organic cages are packed into a one-dimensional supramolecular tube depending on Van der Waals force, which are further packed into a three-dimensional porous supramolecular framework depending on Van der Waals interactions as well. In particular, neither intramolecular nor intermolecular neighboring porphyrin units have been arranged in a face-to-face packing mode in the porous cage-based supramolecular framework, enabling the solid sample to exhibit excellent heterogeneous photocatalytic properties through overcoming the general aggregation-induced photocatalyst deactivation for porphyrin materials. It is worth noting that, due to the isostructural nature of PTC-1(2H) enantiomers, subsequent measurements were carried out only on (*R*)-PTC-1(2H).

### Permanent porosity of PTC-1(2H)

**Fig. 3:**
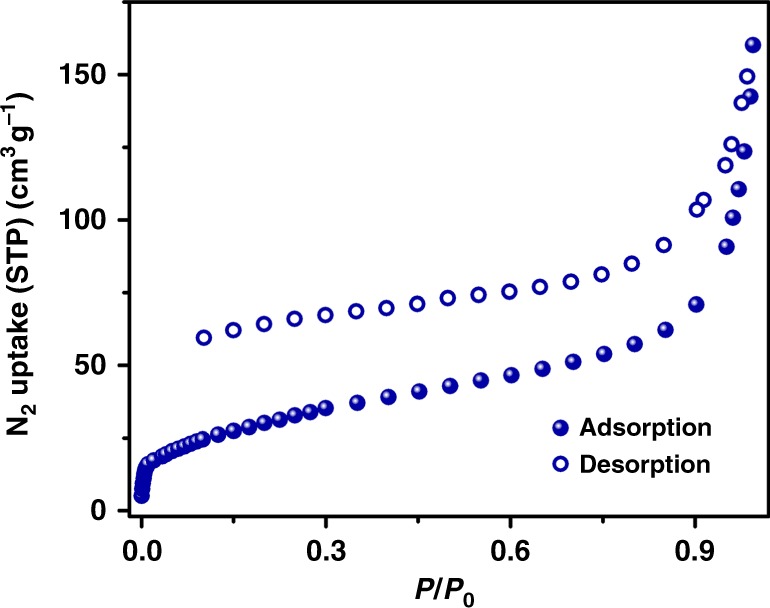
N_2_ sorption isotherms of PTC-1(2H) at 77 K. Source data are provided as a Source Data file.

N_2_ sorption experiment at 77 K was conducted to identify the permanent porosity of cage-based framework. PTC-1(2H) shows a typical type I gas sorption behavior with the Brunauer–Emmett–Teller surface area of 112 m^2^ g^–1^ (Fig. 3). The large pore size of 1.35 and 2.73 nm for PTC-1(2H) would benefit the small molecule diffusion during catalytic process (Supplementary Fig. 14). In addition, the powder X-ray diffraction pattern of the degassed cage-based framework matches well with that of the as-prepared sample, indicating the robust PTC-1(2H) supramolecular framework (Supplementary Fig. 15).

### Photophysical behaviors of PTC-1(2H)

The molecular photophysical behaviors for PTC-1(2H) were studied via electronic absorption, fluorescence, and transient absorption (TA) with monomeric H_2_CBPP as reference. PTC-1(2H) in toluene exhibits a typical electronic absorption spectrum for metal-free porphyrin chromophores with a strong Soret band at 397 nm and four weak Q bands at 506, 542, 580, and 635 nm (Table 1 and Supplementary Fig. 8). Only the Soret band of PTC-1(2H) takes a big blue-shift of 15 nm in comparison with that of H_2_CBPP (412 nm) due to the presence of weak intramolecular C‒H–π interaction as revealed in the structure section. In CH_2_Cl_2_ and dimethylformamide (DMF), the Soret band and four Q bands of PTC-1(2H) and H_2_CBPP almost do not change (Supplementary Fig. 8 and Supplementary Table 3).

Upon the excitation at 405 nm, a broad split emission band peaking at 638 and 702 nm was observed for PTC-1(2H) in toluene with an absolute quantum yield of 1.82% and a lifetime of 9.80 ns (Table 1 and Supplementary Figs. 16–19 and Supplementary Table 3). Both the fluorescence quantum yield and lifetime of PTC-1(2H) are lower than those for H_2_CBPP, namely, 1.86% and 9.87 ns, respectively, due to the presence of much faster non-radiative and intersystem crossing process within the organic cage^57^. Similar phenomenon was also observed in the emission of PTC-1(2H) and H_2_CBPP in CH_2_Cl_2_ and DMF, respectively, Supplementary Table 3.

**Fig. 4:**
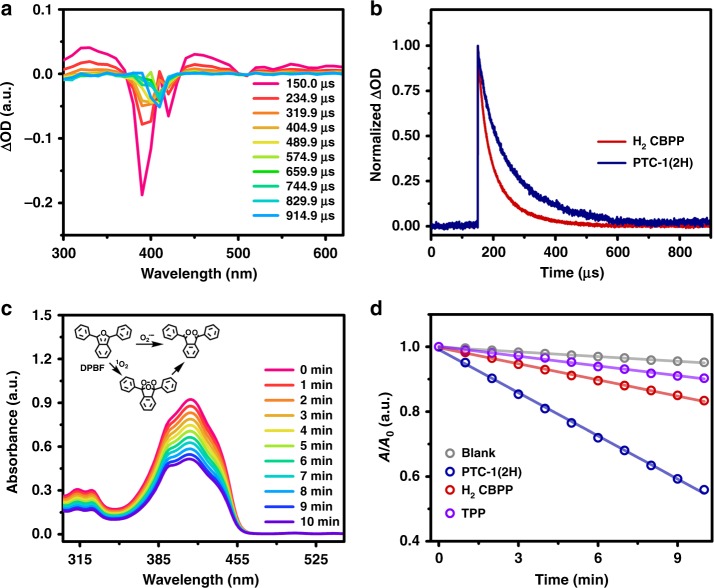
Homogeneous photophysical and photocatalytic behaviors of PTC-1(2H). **a** Nanosecond transient absorption spectra for a toluene solution of PTC-1(2H) excited upon 355 nm laser pulse under nitrogen condition; **b** normalized kinetic traces of the 450 nm band of PTC-1(2H) and H_2_CBPP under nitrogen condition; **c** time-dependent absorption spectra of DPBF in DMF upon visible light irradiation (*λ* > 510 nm) in the presence of PTC-1(2H); **d** comparison of absorbance decay rate at 416 nm of DPBF in the presence of different catalysts. Source data are provided as a Source Data file.

Toward understanding the relaxation process of the organic cage, nanosecond TA spectroscopy under the irradiation of the 355 nm laser pulse was employed. It is worth noting that, before the measurements, the concentration of porphyrin photosensitizers, including PTC-1(2H) and H_2_CBPP reference, was adjusted to keep the same absorption intensity (0.3) at 355 nm. In nitrogen atmosphere, PTC-1(2H) displays the negative absorption band in the range of 370–430 nm due to the typical ground-state bleaching of porphyrin core (Fig. 4a)^10,58^. The positive bands observed at 430–500 nm are attributed to the characteristic porphyrin triplet absorption^10,58^. This is also true for the monomeric reference H_2_CBPP with ground-state bleaching and triplet bands observed at 380–425 and 425–500 nm (Supplementary Fig. 20). It is worth noting that the triplet quantum yield for either PTC-1(2H) or H_2_CBPP could not be quantitatively evaluated owing to the overlapping between their triplet band and ground-state bleaching band, Fig. 4a and Supplementary Fig. 20a^59^. The similar triplet quantum yield for PTC-1(2H) to that of H_2_CBPP, however, is qualitatively unveiled by their similar absolute fluorescence quantum yield and lifetime^60^, while the triplet lifetime (102.20 μs) for this cage is almost twice longer than that of monomer (53.98 μs), which is associated with the much more rigid structure of the cage molecule (Fig. 4b)^61^. This result suggests the existence of much more reaction opportunities between the triplet excited states of cages and ^3^O_2_ molecules, being helpful for the photo-inducing evolution of ROS. The TA spectra of PTC-1(2H) in air are shown (Supplementary Fig. 21). Both ground-state bleaching and triplet absorption bands did not show any obvious shift. The triplet lifetime of PTC-1(2H) got seriously shortened to 0.33 μs, in comparison to that in N_2_, 102.20 μs, due to the reaction occurring between the triplet excited states of porphyrin organic cages and O_2_ dissolved in solution (Table 1 and Supplementary Fig. 22).

### Homogeneous photocatalytic property of PTC-1(2H)

Prior to evaluating their visible light photocatalytic activity toward ROS evolution, the electrochemical behavior for PTC-1(2H) was studied. Electrochemical measurement result reveals the similar reduction potential of −0.97 V for PTC-1(2H) to −0.98 V for H_2_CBPP due to the very weak intracage interaction as disclosed by theoretically calculated frontier molecular orbitals (Table 1 and Supplementary Figs. 23 and 24). The higher reduction potentials of these samples than the reduction potential −0.56 V (vs. standard calomel electrode) for O_2_/O_2_^•‒^ indicate the photocatalytic capacity of organic cage toward generating O_2_^•‒^^62^. This is verified by the subsequent time-dependent photo-bleach experiment based on the oxidation of 1,3-diphenylisobenzofuran (DPBF) by ^1^O_2_ and O_2_^•‒^ using PTC-1(2H) as photocatalyst in DMF solution under the visible light irradiation of *λ* > 510 nm (Fig. 4c and Supplementary Fig. 25)^63^. Nevertheless, PTC-1(2H) shows much higher ROS (actually a total ^1^O_2_ and O_2_^•‒^) generation efficiency than monomeric H_2_CBPP and a typical porphyrin representative of 5,10,15,20-tetraphenylporphyrin (TPP), illustrating the prominent homogeneous photocatalytic property of the cage-like photosensitizer (Fig. 4d). The higher quantum yield of singlet oxygen for PTC-1(2H) (*Φ*_Δ_ = 0.93) than that for H_2_CBPP (*Φ*_Δ_ = 0.87) in toluene was quantitatively measured on the basis of the typical emission with the center at ca. 1270 nm with TPP as reference (*Φ*_Δ_ = 0.70)^64^ (Supplementary Fig. 26). This, in cooperation with the similar quantum yield of triplet state for PTC-1(2H) and H_2_CBPP unveiled in a qualitative manner as detailed above, confirms the crucial role of triplet lifetime in the photo-driven singlet oxygen evolution.

In addition, the electron spin-resonance (ESR) spectroscopy was also employed to further check the photocatalytic activities of PTC-1(2H) with 2,2,6,6-tetramethylpiperidine (TEMP) and 5,5-dimetyl-1-pyrroline N-oxide (DMPO) as ^1^O_2_- and O_2_^•‒^-sensitive probes, respectively^63^. The ESR spectra for either TEMP or DMPO trapping agents in the presence of photosensitizer including PTC-1(2H) and three-equivalent H_2_CBPP kept almost silent before the irradiation of a blue light-emitting diode (LED) light (420 nm < *λ*_em_ < 490 nm) (Supplementary Figs. 27 and 28). The irradiation led to an expected appearance of ESR signals for both TEMP and DMPO capturers, indicating the production of ^1^O_2_ and O_2_^•‒^, respectively. In particular, the ESR signal intensity with the ^1^O_2_ scavenger of TEMP over organic cage is significantly larger than the monomer-induced signal, confirming the outstanding visible light-driven homogeneous photocatalytic properties of PTC-1(2H) in singlet oxygen evolution associated with the long triplet lifetime. In contrast, the similar DMPO-O_2_^•‒^ ESR signal intensity monitored after addition of organic cage or monomer in a three-equivalent amount indicates their comparable visible light photocatalytic performance in terms of the O_2_^•‒^ evolution (Supplementary Fig. 28). As a total result, the cage triplet lifetime rather than the triplet state quantum yield dominates the present visible light-induced generation of ROS (mainly the singlet oxygen). This result must be helpful in the application of molecular cage in the field of photodynamic therapy and artificial photosynthesis.

Further evaluating the photocatalytic properties of PTC-1(2H), the oxidation coupling reaction of benzylamine with ^1^O_2_ and O_2_^•‒^ was studied under a blue LED light irradiation. In fact, selective oxidation of amine is of significance in the synthesis of biologically active imine derivatives^65^. With the help of cage-based photocatalyst and within a short time of ca. 15.0 min, >99% benzylamine molecules have been converted into *N*-benzylidenebenzylamine (based on the NMR data; Table 2). In contrast, a longer reaction time of 20.0 and 25.0 min becomes necessary for the monomer photocatalysts, H_2_CBPP and TPP, respectively, to convert >99% benzylamine molecules to target product. This, in cooperation with the TA and ESR spectroscopic results as mentioned above, confirms not only the prominent photocatalytic performance of organic cage under visible light irradiation but also the long triplet lifetime-related promotion mechanism. In the control experiments with reaction exposed in air and in the absence of PTC-1(2H) photocatalyst, the conversion of benzylamine into *N*-benzylidenebenzylamine either proceeded in much slower speed or kept unreacted, illustrating the crucial role of molecular cage-based photocatalyst and oxygen. Nevertheless, the homogeneous control experiments in the presence of 5.00 μmol triethylenediamine (DABCO) as an efficient singlet oxygen quencher and 5.00 μmol benzoquinone (BQ) as a trapping agent for O_2_^•‒^ gave 9% and 90% conversion of benzylamine to the corresponding product, respectively (Table 2), indicating the more important role of singlet oxygen instead of O_2_^•‒^ in the present reaction process.

**Table 2:** Homogeneous visible light oxidative coupling of various amines into imines^a^.


Entry	Catalyst	Atmosphere	*t* (min)	Conv. (%)^b^	TOF^c^
1	—	O_2_	15.0	Trace	—
2	PTC-1(2H)	O_2_	15.0	>99	1320
3	PTC-1(2H)	Air	20.0	>99	990
4	PTC-1(2H)	N_2_	15.0	Trace	—
5^d^	PTC-1(2H)	O_2_	15.0	Trace	—
6^e^	H_2_CBPP	O_2_	20.0	>99	990
7^f^	TPP	O_2_	25.0	>99	790
8^g^	PTC-1(2H)	O_2_	15.0	10	130
9^h^	PTC-1(2H)	O_2_	15.0	90	1200

^a^0.10 mmol benzylamine, 0.10 μmol PTC-1(2H), 1.0 atm O_2_, 25 W blue LED (420 nm < *λ* < 490 nm), and 0.5 mL toluene-*d*_8_.

^b^Determined by ^1^H NMR analysis every 5 min.

^c^TOF value was calculated by mmol of amine converted per mmol of porphyrin unit per hour.

^d^No irradiation.

^e^3.00 μmol H_2_CBPP.

^f^3.00 μmol TPP.

^g^0.10 μmol PTC-1(2H) and 5.00 μmol triethylenediamine (DABCO).

^h^0.10 μmol PTC-1(2H) and 5.00 μmol benzoquinone (BQ).

### Heterogeneous photocatalytic properties of PTC-1(2H)

**Fig. 5:**
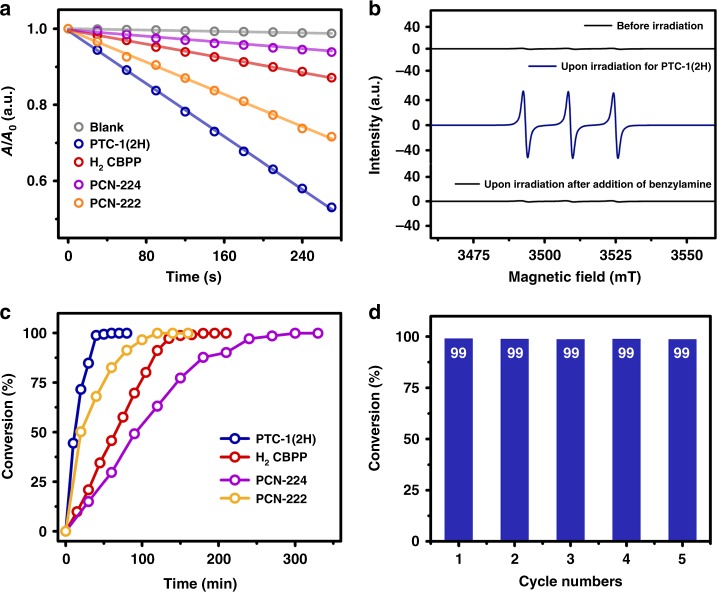
Heterogeneous photocatalytic behavior of PTC-1(2H). **a** Comparison of absorbance decay rate at 410 nm of DPBF in the presence of different photocatalysts in CH_3_CN upon visible light irradiation of *λ* > 510 nm; **b** ESR detection of ^1^O_2_ generation over PTC-1(2H) trapped by TEMP in CH_3_CN; **c** comparison of time-dependent heterogeneous catalytic efficiency of different photocatalysts; **d** recycle test of PTC-1(2H) in the photo-induced oxidative coupling of benzylamine to *N*-benzylidenebenzylamine. Source data are provided as a Source Data file.

It is well known that the practical application of the molecular porphyrins as heterogeneous photocatalysts has been retarded by the aggregation-induced deactivation^13,15^. The lack of both the intramolecular and intermolecular face-to-face packed porphyrin moieties in the cage-based supramolecular framework as revealed by single crystal X-ray analysis, in combination with the existence of moderately permanent porosity in the PTC-1(2H) framework, therefore inspires our interest toward investigating its heterogeneous photocatalytic performance. For this purpose, the visible light-induced ROS evolution capability of crystalline PTC-1(2H) sample in acetonitrile was examined first. For comparative studies, the ROS evolution activities of solid H_2_CBPP and two well-known porphyrin-containing MOFs of PCN-222 (also known as MOF-545 or MMPF-6)^66,67^ and PCN-224^68^ were also examined under the same concentration of porphyrin units and experimental conditions. The visible light-induced DPBF photo-bleach time-dependent curves reveal the highest ROS generation efficiency of the solid PTC-1(2H), verifying the expected excellent heterogeneous photocatalytic property of this cage-based supramolecular framework (Fig. 5a and Supplementary Fig. 29).

In addition, visible light-induced photo-oxidation of benzylamine by oxygen was chosen again as a heterogeneous catalytic model reaction. In the oxygen atmosphere, >99% benzylamine in CD_3_CN could be converted into *N*-benzylidenebenzylamine within only 60 min with the help of PTC-1(2H) supramolecular framework (Table 3). ESR spectroscopic data with TEMP and DMPO sensor, respectively, indicate the seriously reduced intensity of photo-induced signals after addition of benzylamine into the suspension of PTC-1(2H) supramolecular framework in CD_3_CN, disclosing the association of high-efficiency conversion of benzylamine with ^1^O_2_ and O_2_^•‒^ (Fig. 5b and Supplementary Fig. 30)^64^. The heterogeneous control experiments in the presence of the same amount of DABCO and BQ gave 25% and 57% conversion of benzylamine, respectively (Table 3), further revealing the more important role of singlet oxygen in the present conversion upon cage photocatalyst. In the control experiments, much longer reaction time of 180, 120, and 300 min were needed for solid H_2_CBPP, PCN-222, and PCN-224, respectively, instead of PTC-1(2H) as heterogeneous photocatalyst to achieve >99% conversion of benzylamine (Fig. 5c and Table 3), illustrating the best heterogeneous photocatalytic activity of PTC-1(2H) supramolecular framework among these species.

**Table 3:** Heterogeneous visible light oxidative coupling of various amines into imines^a^.


Entry	Substrate	Product	*t* (min)	Conv. (%)^b^	TOF^c^
1	R=H	R=H	60	>99	33.0
2	R=F	R=F	50	>99	39.6
3	R=CH_3_	R=CH_3_	60	>99	33.0
4	R=OCH_3_	R=OCH_3_	70	>99	28.2
5	R=Cl	R=Cl	90	>99	22.0
6			50	>99	39.6
7			120	>99	16.5
8			140	>99	14.1
9^d^	R=H	R=H	180	>99	11.0
10^e^	R=H	R=H	120	>99	16.5
11^f^	R=H	R=H	300	>99	6.6
12^g^	R=H	R=H	60	25	8.3
13^h^	R=H	R=H	60	57	19.0

^a^Reaction conditions: 0.10 mmol substrate, 1.00 μmol PTC-1(2H) photocatalyst, 1.0 mL CD_3_CN, 1.0 atm O_2_, 25 W blue LED (420 nm < *λ*_em_ < 490 nm).

^b^Determined based on ^1^H NMR data.

^c^TOF value was calculated by mmol of amine converted per mmol of porphyrin unit per hour.

^d^3.00 μmol H_2_CBPP.

^e^3.00 μmol PCN-222.

^f^3.00 μmol PCN-224.

^g^1.00 μmol PTC-1(2H) and 10.00 μmol DABCO.

^h^1.00 μmol PTC-1(2H) and 10.00 μmol BQ.

After the conversion of benzylamine, the PTC-1(2H) catalyst was separated from the reaction mixture in CD_3_CN and washed by *n*-hexane for three times to remove the residue chemical over the catalyst surface. ^1^H NMR spectroscopic results recorded in CD_3_CN containing dispersed PTC-1(2H) catalyst then clearly reveal the release of *N*-benzylidenebenzylamine from the pores of the heterogeneous catalyst (Supplementary Fig. 31), indicating the effective diffusion of product molecule from the pores within PTC-1(2H) framework due to the big pore size. This might preclude the product inhibition effect^49^ and benefit the photo-induced benzylamine conversion. Nevertheless, the highly efficient and constant conversion of benzylamine under the photocatalysis of PTC-1(2H) framework continues for five times, demonstrating the good recyclability of solid PTC-1(2H) catalyst (Fig. 5d).

For the purpose of revealing the general applicability of PTC-1(2H) heterogeneous photocatalyst, the photo-oxidation of a series of primary amine derivatives was carried out. With the assistance of PTC-1(2H) supramolecular framework, the whole series of benzylamine derivatives substituted with different functional groups could be converted to corresponding products with >99% conversion in a specified period, revealing the excellent properties of solid porphyrin organic cage PCT-1(2H) as a heterogeneous photocatalyst (Table 3). Among the diverse substrates, (4-fluorophenyl)methanamine converted to *N*-(4-fluorobenzyl)-1-(4-fluorophenyl)methanimine within the shortest time of 50 min due to the electron-withdrawing effect of fluorine atom. This is also true for 3,4-difluorobenzylamine. In contrast, much longer time ranging from 90 and 120 min becomes necessary for the complete conversion of the chlorine-substituted substrates including 4-chorobenzylamine and 3,4-dichorobenzylamine to corresponding *N*-benzylidenebenzylamine derivatives due to the effect of the *p*–π conjugation for chlorine atom^69^.

## Discussion

A [3+6] metal-free porphyrin organic cage in a tubular structure with the length of 3.3 nm has been successfully fabricated and assembled into porous supramolecular framework. The cage-induced long triplet lifetime was revealed to play crucial role in the high-efficiency singlet oxygen evolution and in turn the photo-oxidation of benzylamine in homogeneous phase. More importantly, the porphyrin cage-based porous supramolecular framework overcomes the general aggregation-induced deactivation of molecular porphyrins as heterogeneous catalyst and efficiently promotes photo-oxidation coupling reaction of various primary amines. The present result discloses the unique catalytic activity of porous organic cages in heterogeneous photocatalysis associated with the unique cage-induced long triplet lifetime and porous structure, which is expected to ignite more research interest toward exploration of porous organic cage with great heterogeneous application potentials.

## Methods

### Remark

Dichloromethane was freshly distilled from CaH_2_. The other commercial chemicals were used without any further treatment. H_2_DBPP was prepared from the reaction between 5,15-bis[4-(4,4,5,5-tetramethyl-1,3,2-dioxaborolan-2-yl)phenyl]porphyrin^70^ and 5-bromoisophthalaldehyde. For details, please see Supplementary Information.

### Synthesis of PTC-1(2H)

To a suspension of H_2_DBPP (0.06 mmol) and trifluoroacetic acid (2.0 μL) in dichloromethane (50 mL), a solution of enantiomerically pure cyclohexanediamine (14.8 mg, 0.130 mmol) in dichloromethane (20 mL) was added slowly. The mixture was stirred at room temperature for 24 h. Then the reaction mixture was evaporated under reduced pressure and applied on a silica gel column using tetrahydrofuran as eluent. Repeated column chromatography followed by recrystallization in chloroform and methanol gave the target organic cages as dark red powder in the yield of 85.6% and 87.3% for (*R*)-PTC-1(2H) and (*S*)-PTC-1(2H), respectively. ^1^H NMR (400 MHz, CDCl_3_): *δ* 8.78 (d, *J* = 4.5 Hz, 12H), 8.64 (d, *J* = 16.3 Hz, 12H), 8.54 (s, 6H), 8.37 (d, *J* = 7.6 Hz, 12H), 8.23 (s, 6H), 8.16 (d, *J* = 7.7 Hz, 12H), 8.07 (s, 6H), 7.75–7.45 (m, 18H), 3.71 (dd, *J* = 14.0, 8.8 Hz, 6H), 3.57 (dd, *J* = 15.4, 8.5 Hz, 6H), 2.00 (d, *J* = 11.8 Hz, 24H), 1.70 (s, 24H), -3.56 (s, 6H); ^13^C NMR (100 MHz, CDCl_3_): *δ* 160.67, 160.31, 146.71, 144.34, 141.13, 141.02, 139.18, 137.44, 137.19, 135.18, 132.51, 131.32, 130.04, 125.63, 118.11, 104.50, 75.66, 74.99, 33.27, 32.81, 29.70, 24.68; MS (MALDI-TOF) *m*/*z*: [M]+calcd. for C_180_H_150_N_24_, 2649.25; found: 2649.53 for (*R*)-PTC-1(2H) and 2649.34 for (*S*)-PTC-1(2H); analysis of (*R*)-PTC-1(2H) (calcd., found for C_180_H_150_N_24_·1.5CHCl_3_): C (77.07, 76.96), H (5.40, 5.88), N (11.89, 11.66); analysis of (*S*)-PTC-1(2H) (calcd., found for C_180_H_150_N_24_): C (81.60, 81.22), H (5.70, 5.44), N (12.69, 12.87).

## Supplementary information

Supplementary Information

Description of Additional Supplementary Files

Supplementary Data 1

## Data Availability

The authors declare that the data supporting the findings of this study are available within the main manuscript and Supplementary Information. The source data underlying Figs. 3, 4a–c, and 5b, c and Supplementary Figs. 7–9, 14–22, 25, and 27–30 are provided as a Source Data file. Extra data are available from the corresponding author upon reasonable request. The crystallographic data in this study have been deposited in the Cambridge Structural Database under entry IDs CCDC 1913971 and 1913972. These data can be obtained free of charge from The Cambridge Crystallographic Data Centre via www.ccdc.cam.ac.uk/data_request/cif.

## Source Data

Source Data

## References

[1] Fagnoni M, Dondi D, Ravelli D, Albini A (2007). Photocatalysis for the formation of the C−C bond. Chem. Rev..

[2] Hoffmann N (2008). Photochemical reactions as key steps in organic synthesis. Chem. Rev..

[3] Ravelli D, Fagnoni M, Albini A (2013). Photoorganocatalysis. what for?. Chem. Soc. Rev..

[4] Prier CK, Rankic DA, MacMillan DWC (2013). Visible light photoredox catalysis with transition metal complexes: applications in organic synthesis. Chem. Rev..

[5] Schultz DM, Yoon TP (2014). Solar synthesis: prospects in visible light photocatalysis. Science.

[6] Knoll JD, Albani BA, Turro C (2015). New Ru(II) complexes for dual photoreactivity: ligand exchange and ^1^O_2_ generation. Acc. Chem. Res..

[7] Ladomenou K (2015). Photochemical hydrogen generation with porphyrin-based systems. Coord. Chem. Rev..

[8] Urbani M, de la Torre G, Nazeeruddin MK, Torres T (2019). Phthalocyanines and porphyrinoid analogues as hole- and electron-transporting materials for perovskite solar cells. Chem. Soc. Rev..

[9] Ke X-S (2014). Porphodilactones as synthetic chlorophylls: relative orientation of β-substituents on a pyrrolic ring tunes NIR absorption. J. Am. Chem. Soc..

[10] Qi Q (2019). Preferential binding of π-ligand porphyrin targeting 5′-5′ stacking interface of human telomeric RNA G-quadruplex dimer. J. Phys. Chem. Lett..

[11] Lu H, Kobayashi N (2016). Optically active porphyrin and phthalocyanine systems. Chem. Rev..

[12] Dąbrowski JM (2016). Engineering of relevant photodynamic processes through structural modifications of metallotetrapyrrolic photosensitizers. Coord. Chem. Rev..

[13] Xiao J-D, Jiang H-L (2019). Metal–organic frameworks for photocatalysis and photothermal catalysis. Acc. Chem. Res..

[14] He W-L, Zhao M, Wu C-D (2019). A versatile metalloporphyrinic framework platform for highly efficient bioinspired, photo- and asymmetric catalysis. Angew. Chem. Int. Ed..

[15] Liu Y, Howarth AJ, Hupp JT, Farha OK (2015). Selective photooxidation of a mustard-gas simulant catalyzed by a porphyrinic metal–organic framework. Angew. Chem. Int. Ed..

[16] Ding X, Han B-H (2015). Metallophthalocyanine-based conjugated microporous polymers as highly efficient photosensitizers for singlet oxygen generation. Angew. Chem. Int. Ed..

[17] Das MC, Xiang S, Zhang Z, Chen B (2011). Functional mixed metal–organic frameworks with metalloligands. Angew. Chem. Int. Ed..

[18] Chen R (2019). Designed synthesis of a 2D porphyrin-based sp^2^ carbon-conjugated covalent organic framework for heterogeneous photocatalysis. Angew. Chem. Int. Ed..

[19] Tranchemontagne DJ, Ni Z, O’Keeffe M, Yaghi OM (2008). Reticular chemistry of metal–organic polyhedra. Angew. Chem. Int. Ed..

[20] Saha ML, Yan X, Stang PJ (2016). Photophysical properties of organoplatinum(ii) compounds and derived self-assembled metallacycles and metallacages: fluorescence and its applications. Acc. Chem. Res..

[21] Li J-R, Zhou H-C (2010). Bridging-ligand-substitution strategy for the preparation of metal–organic polyhedra. Nat. Chem..

[22] Zhang D, Ronson TK, Nitschke JR (2018). Functional capsules via subcomponent self-assembly. Acc. Chem. Res..

[23] Jing X, He C, Zhao L, Duan C (2019). Photochemical properties of host–guest supramolecular systems with structurally confined metal–organic capsules. Acc. Chem. Res..

[24] Chen L, Chen Q, Wu M, Jiang F, Hong M (2015). Controllable coordination-driven self-assembly: from discrete metallocages to infinite cage-based frameworks. Acc. Chem. Res..

[25] Gong W (2019). Permanent porous hydrogen-bonded frameworks with two types of Brønsted acid sites for heterogeneous asymmetric catalysis. Nat. Commun..

[26] Yan X, Cook TR, Wang P, Huang F, Stang PJ (2015). Highly emissive platinum(II) metallacages. Nat. Chem..

[27] Hasell T, Cooper AI (2016). Porous organic cages: soluble, modular and molecular pores. Nat. Rev. Mater..

[28] Ke X-S (2018). Three-dimensional fully conjugated carbaporphyrin cage. J. Am. Chem. Soc..

[29] Mastalerz M (2018). Porous shape-persistent organic cage compounds of different size, geometry, and function. Acc. Chem. Res..

[30] Qu H (2017). Molecular face-rotating cube with emergent chiral and fluorescence properties. J. Am. Chem. Soc..

[31] Han B (2019). Postsynthetic metalation of a robust hydrogen-bonded organic framework for heterogeneous catalysis. J. Am. Chem. Soc..

[32] Mukhopadhyay RD, Kim Y, Koo J, Kim K (2018). Porphyrin boxes. Acc. Chem. Res..

[33] Jones JTA (2011). Modular and predictable assembly of porous organic molecular crystals. Nature.

[34] Bera S (2019). Porosity switching in polymorphic porous organic cages with exceptional chemical stability. Angew. Chem. Int. Ed..

[35] Smith PT (2018). Iron porphyrins embedded into a supramolecular porous organic cage for electrochemical CO_2_ reduction in water. Angew. Chem. Int. Ed..

[36] Hong S (2015). Porphyrin boxes: rationally designed porous organic cages. Angew. Chem. Int. Ed..

[37] Wang Z (2019). Soft porous crystal based upon organic cages that exhibit guest-induced breathing and selective gas separation. J. Am. Chem. Soc..

[38] Liu Y, Hu C, Comotti A, Ward MD (2011). Supramolecular archimedean cages assembled with 72 hydrogen bonds. Science.

[39] Rebek J (2009). Molecular behavior in small spaces. Acc. Chem. Res..

[40] Omagari T, Suzuki A, Akita M, Yoshizawa M (2016). Efficient catalytic epoxidation in water by axial n-ligand-free Mn-porphyrins within a micellar capsule. J. Am. Chem. Soc..

[41] Shi Y (2018). Selective extraction of C70 by a tetragonal prismatic porphyrin cage. J. Am. Chem. Soc..

[42] Cremers J (2018). Template-directed synthesis of a conjugated zinc porphyrin nanoball. J. Am. Chem. Soc..

[43] Benke BP (2017). Iodide-selective synthetic ion channels based on shape-persistent organic cages. J. Am. Chem. Soc..

[44] Ward MD, Hunter CA, Williams NH (2018). Coordination cages based on bis(pyrazolylpyridine) ligands: structures, dynamic behavior, guest binding, and catalysis. Acc. Chem. Res..

[45] Oliveri CG (2006). Supramolecular allosteric cofacial porphyrin complexes. J. Am. Chem. Soc..

[46] Fiedler D, Leung DH, Bergman RG, Raymond KN (2005). Selective molecular recognition, C−H bond activation, and catalysis in nanoscale reaction vessels. Acc. Chem. Res..

[47] Inokuma Y (2013). X-ray analysis on the nanogram to microgram scale using porous complexes. Nature.

[48] Iwasawa T, Hooley RJ, Rebek J (2007). Stabilization of labile carbonyl addition intermediates by a synthetic receptor. Science.

[49] Yoshizawa M, Tamura M, Fujita M (2006). Diels-alder in aqueous molecular hosts: unusual regioselectivity and efficient catalysis. Science.

[50] Pluth MD, Bergman RG, Raymond KN (2007). Acid catalysis in basic solution: a supramolecular host promotes orthoformate hydrolysis. Science.

[51] Wang Q-Q (2016). Self-assembled nanospheres with multiple endohedral binding sites pre-organize catalysts and substrates for highly efficient reactions. Nat. Chem..

[52] Yang X, Sun J-K, Kitta M, Pang H, Xu Q (2018). Encapsulating highly catalytically active metal nanoclusters inside porous organic cages. Nat. Catal..

[53] Kim Y (2018). Rational design and construction of hierarchical superstructures using shape-persistent organic cages: porphyrin box-based metallosupramolecular assemblies. J. Am. Chem. Soc..

[54] Pan M, Wu K, Zhang J-H, Su C-Y (2019). Chiral metal–organic cages/containers (MOCs): from structural and stereochemical design to applications. Coord. Chem. Rev..

[55] Zhang Z (2019). Aqueous platinum(ii)-cage-based light-harvesting system for photocatalytic cross-coupling hydrogen evolution reaction. Angew. Chem. Int. Ed..

[56] Slater AG (2016). Reticular synthesis of porous molecular 1D nanotubes and 3D networks. Nat. Chem..

[57] Miao X (2019). Deciphering the intersystem crossing in near-infrared BODIPY photosensitizers for highly efficient photodynamic therapy. Chem. Sci..

[58] Andréasson J, Kajanus J, Mårtensson J, Albinsson B (2000). Triplet energy transfer in porphyrin dimers: comparison between π- and σ-chromophore bridged systems. J. Am. Chem. Soc..

[59] Zhang X-F, Yang X, Xu B (2017). PET-based bisBODIPY photosensitizers for highly efficient excited triplet state and singlet oxygen generation: tuning photosensitizing ability by dihedral angles. Phys. Chem. Chem. Phys..

[60] Bachilo SM, Weisman RB (2000). Determination of triplet quantum yields from triplet-triplet annihilation fluorescence. J. Phys. Chem. A.

[61] Šolomek T (2017). Electron hopping and charge separation within a naphthalene-1,4:5,8-bis(dicarboximide) chiral covalent organic cage. J. Am. Chem. Soc..

[62] Ma L (2019). Ferrocene-linkage-facilitated charge separation in conjugated microporous polymers. Angew. Chem. Int. Ed..

[63] Luo J, Lu J, Zhang J (2018). Carbazole–triazine based donor–acceptor porous organic frameworks for efficient visible-light photocatalytic aerobic oxidation reactions. J. Mater. Chem. A.

[64] Shao W (2012). Photophysical properties and singlet oxygen generation of three sets of halogenated corroles. J. Phys. Chem. B.

[65] Murahashi S-I (1995). Synthetic aspects of metal-catalyzed oxidations of amines and related reactions. Angew. Chem. Int. Ed..

[66] Feng D (2012). Zirconium-metalloporphyrin PCN-222: mesoporous metal–organic frameworks with ultrahigh stability as biomimetic catalysts. Angew. Chem. Int. Ed..

[67] Morris W (2012). Synthesis, structure, and metalation of two new highly porous zirconium metal–organic frameworks. Inorg. Chem..

[68] Feng D (2013). Construction of ultrastable porphyrin Zr metal–organic frameworks through linker elimination. J. Am. Chem. Soc..

[69] Fickling MM, Fischer A, Mann BR, Packer J, Vaughan J (1959). Hammett substituent constants for electron-withdrawing substituents: dissociation of phenols, anilinium ions and dimethylanilinium ions. J. Am. Chem. Soc..

[70] Senge MO (2010). Synthesis of meso-substituted ABCD-type porphyrins by functionalization reactions. Eur. J. Org. Chem..

