# Supplementary material for: Elucidating heterogeneous photocatalytic superiority of microporous porphyrin organic cage
Source: Nat Commun. 2020 Feb 26;11:1047. doi: 10.1038/s41467-020-14831-x (PMC7044162; doi:10.1038/s41467-020-14831-x)
Supplement: Supplementary file 1 — Supplementary Information [file 41467_2020_14831_MOESM1_ESM.pdf]

Supplementary Information for

Elucidating heterogeneous photocatalytic superiority of microporous porphyrin organic cage

Liu et al.

Supplementary Methods

General remark. Dichloromethane was freshly distilled from CaH_2. The other commercial chemicals were used without any treatment. 5,15-Bis[4-(4,4,5,5-tetramethyl-1,3,2-dioxaborolan-2-yl)phenyl]porphyrin (H_2BTPP).[1]

Synthesis of 5,15-bis[4-(4,4,5,5-tetramethyl-1,3,2-dioxaborolan-2-yl)-phenyl]porphyrin (H_2BTPP). To a mixture of dipyrromethane (675 mg, 4.62 mmol), 4-(4,4,5,5-tet-ramethyl-1,3,2-dioxaborolan-2-yl)benzaldehyde (1.12 g, 4.86 mmol), and dichloromethane (500 mL) in a 1000.0 mL three-necked flask, TFA (50.0 mg, 0.462 mmol) was slowly added in 60 min. The mixture was stirred for 12 h under nitrogen atmosphere. Then 2,3-dicyano-5,6-dichlorobenzoquinone (2.10 g, 9.25 mmol) was added to the reaction mixture. After continuously stirring for another 90.0 min at room temperature, trimethylamine (1.0 mL) was added to quench the reaction. The crude reaction mixture was directly chromatographed on a silica gel (200-300 mesh) column using dichloromethane as eluent. The solvent was evaporated in vacuum, and the resulting residue was recrystallized from dichloromethane and methanol, giving a pure purple product (250 mg) in a yield of 15.2%. ^{1}H NMR (400 MHz, $CDCl_3$): δ 10.32 (s, 2H), 9.39 (s, 4H), 9.07 (s, 4H), 8.27 (d, J = 10.3 Hz, 8H), 1.54 (s, 24H), -3.12 (s, 2H).

Synthesis of metal-free 5,15-di[3′,5′-diformyl-(1,1′-biphenyl)]porphyrin (H_2DBPP). A mixture of H_2BTPP (143 mg, 0.200 mmol), 5-bromoisophthalaldehyde (94.0 mg, 0.440 mmol), potassium carbonate (552 mg, 4.00 mmol), tetrakis(triphenylphosphine)palladium (58.0 mg, 50.0 μmol), tetrahydrofuran (18.0 mL), and deionized water (2.0 mL) in a 50.0 mL flask was stirred at 90°C for 36 hours. The reaction mixture was cooled to room temperature and filtered. The crude product

was washed with deionized water (10.0 mL), tetrahydrofuran (10.0 mL), and methanol (10.0 mL), respectively, and finally dried at room temperature to afford H_2DBPP (121 mg) in a yield of 83.4%. Because H_2DBPP is insoluble in common organic solvents (such as DMSO, DMF, CH_2Cl_2, $CHCl_3$, and CH_3OH), it is hard to get its ^{1}H NMR spectrum. As a consequence, H_2DBPP was directly used in the next experiment.

Synthesis of metal-free 5,15-di[3',5'-cyclohexyliminomethyl-1,1'-biphenyl]porphyrin (H_2CBPP). To a stirred dichloromethane (50.0 mL) suspension of H_2DBPP (44.0 mg, 60.0 μmol) and trifluoroacetic acid (TFA, 2.0 μL), a solution of cyclohexylamine (26.2 mg, 0.260 mmol) in dichloromethane (10.0 mL) was added. The mixture was stirred for 24 hours at room temperature. Then, the reaction mixture was evaporated in vacuum and chromatographed on a silica gel column using dichloromethane as eluent. The product (46.0 mg) was obtained by recrystallization of crude product in chloroform and methanol. ^{1}H NMR (400 MHz, $CDCl_3$): δ 10.34 (s, 2H), 9.43 (d, J = 4.5 Hz, 4H), 9.15 (d, J = 4.5 Hz, 4H), 8.57 (s, 4H), 8.47–8.29 (m, 8H), 8.20–8.05 (m, 6H), 3.34 (t, J = 10.4 Hz, 4H), 1.95–1.81 (m, 16H), 1.71 (dd, J = 23.6, 10.8 Hz, 12H), 1.50–1.26 (m, 12H), –3.05 (s, 2H); ^{13}C NMR ($CDCl_3$, 100 MHz): δ 158.20, 147.19, 145.28, 141.78, 140.84, 139.67, 137.86, 135.27, 131.76, 131.00, 128.60, 127.40, 125.97, 118.68, 105.40, 70.14, 34.47, 25.72, 24.89; MS (MALDI-TOF) *m/z*: $[M+H]^+$ calcd for $C_{72}H_{74}N_8$, 1051.61; found: 1051.01; analysis of H_2CBPP (calcd., found for $C_{72}H_{74}N_8$): C (82.25, 82.35), H (7.09, 7.21), N (10.66, 10.62).

Characterizations. ^{1}H NMR spectra were recorded on a Bruker DPX 400 spectrometer in $CDCl_3$, CD_3CN, and toluene-d_8 with the residual solvent resonances (δ = 7.26 ppm for $CDCl_3$, δ = 1.94 ppm for CD_3CN, and δ = 2.08 ppm for toluene-d_8)

relative to $SiMe_4$ as internal reference at 298 K and 400 MHz. ^{13}C NMR spectra were recorded by means of the same equipment and referenced internally using the solvent resonance (δ = 53.84 ppm for $CDCl_3$) at 298 K and 100 MHz. Elemental analysis was performed on an Elementar Vavio El III. The electronic absorption spectra were collected using a Perkin-Elmer Lambda 750 UV-vis spectrophotometer. Cyclic voltammetry measurements were carried out on a CHI 760E electrochemical workstation (Chenhua Instrument, Shanghai, China) in a standard three-electrode system. CD spectra were determined using a JASCO J-815 CD spectropolarimeter. Steady-state and transient-state emission spectra together with singlet oxygen quantum yields were recorded on an Edinburgh FLS 980 instrument, and absolute fluorescence quantum yields was measured by using a calibrated integrating sphere system on this instrument. The thermogravimetric analysis (TGA) was performed on a Perkin-Elmer instrument over the temperature range of 25 to 800ºC under nitrogen atmosphere with a heating rate of 10°C/min. The nanosecond time-resolved laser flash photolysis was used to measure the decay kinetics of cage and monomer. In brief, an LP980 spectrometer (Edinburgh Instruments, UK) is synchronized with a commercial Nd:YAG laser (Lab 170, Spectral Physics Inc.). Laser pulse (355 nm) (1 Hz, fwhm $\approx$ 7 ns, 20 mJ/pulse) was used to irradiate the sample solution in 1 cm thickness quartz cuvettes. A 150 W pulsed xenon lamp was used as the probe light. A photomultiplier (PMT) comprising a monochromator was used to collect the kinetic traces at certain absorption wavelengths. The data were analyzed and fitted by L900 software of the LP980 spectrophotometer. It is noteworthy that, before measurement, both solutions of H_2CBPP and PTC-1(2H) in toluene with absorption intensity kept at 0.3 for 355 nm were freshly prepared and saturated with nitrogen for each measurement. ESR measurements were performed at room temperature on Bruker Elexsys E580 X-band.

Powder X-ray diffraction (PXRD) data were collected with a TTR III multi-function X-ray diffractometer operated at 40 kV and 300 mA with Cu Kα radiation. The nitrogen adsorption and desorption isotherms were measured at 77 K using a Micromeritics ASAP 2020 PLUS HD88 system with the samples degassed at room temperature for 24 hours before the measurement.

Single crystal crystallography. Crystallographical data of two organic cages were collected on a diffractometer of SuperNova, Dual, Cu at home/near, AtlasS2 with Cu *Kα* radiation (λ = 1.54184 Å) at 150.00 K. The structures were solved by the direct method (*SHELXS-2014*) and refined by full-matrix least-squares (*SHELXL-2014*) on F^2.[2] Anisotropic thermal parameters were used for the non-hydrogen atoms and isotropic parameters for the hydrogen atoms. Hydrogen atoms were added geometrically and refined using a riding model. Crystallographic and refinement parameters for organic cages are compiled (Supplementary Table 3). Because there are seriously disordered solvent molecules in the cage pores, 'SQUEEZE' command was employed. CCDC 1913971 and 1913972 for (*R*)-PTC-1(2H) and (*S*)-PTC-1(2H), respectively, contain the supplementary crystallographic data for this paper. These data can be obtained free of charge from the Cambridge Crystallographic Data Centre via www.ccdc.cam.ac.uk/data_request/cif.

Cyclic voltammetry measurements. The cell comprised inlets for a glassy-carbocn-disk working electrode with a diameter of 2.00 mm in diameter and a silver-ware counter electrode. The reference electrode was Ag^+/Ag (a solution of 10.0 mM $AgNO_3$ and 0.100 M TBAP in acetonitrile), which was connected to the solution by a Luggin capillary whose tip was placed close to the working electrode. It was corrected

for junction potentials by being referenced internally to the ferrocenium/ferrocene (Fc^+/Fc) couple [$E_{1/2}$(Fc^+/Fc) = 0.501 V *vs*. SCE]. Typically, a 0.100 M solution of [NBu_4][ClO_4] in CH_2Cl_2 containing 1.00 mM of sample was purged with nitrogen for 10.0 min, and then the voltammograms were recorded at ambient temperature. The scan rate was 50.0 mV/s for the CV measurement.

Photo-bleach experiments based on the oxidation of 1,3-diphenylisobenzofuran (DPBF). Comparative study in the reactive oxygen species (ROS) generation of H_2CBPP, PTC-1(2H), TPP, PCN-222, and PCN-224 as either homogeneous photocatalyst or heterogeneous photocatalyst was performed with 1,3-diphenylisobenzofuran (DPBF, 40.0 μmol L^{-1}) as probe with a tungsten lamp ($\lambda > 510$ nm, 10.0 mW cm^{-2}). It is worth noting that, before the measurements, the porphyrin unit concentration of all photosensitizers was kept as 3.00 and 0.300 μmol L^{-1} in acetonitrile and DMF phase, respectively. The time-dependent electronic absorption spectra of DPBF were recorded per 1.0 and 0.5 min for homogeneous and heterogeneous phase, respectively.

Electron spin-resonance (ESR) trapping measurements. For homogeneous phase measurement, a toluene solution of photosensitizer (90.0 μL) with the porphyrin unit concentration of 0.6 mmol L^{-1} was added the toluene solution (10.0 μL) of DMPO (0.400 mol L^{-1}) as $O_2^{\bullet-}$ probe and TEMP (6.00 mol L^{-1}) as $^1O_2$ sensor, respectively. Then, the resulting mixture was transferred to an ESR tube. For heterogeneous phase measurement, photosensitizer (1.00 μmol of PTC-1 or 3.00 μmol reference monomer) was added into the acetonitrile suspension (500 μL) of DMPO (40.0 mmol L^{-1}) and TEMP (0.600 mol L^{-1}), respectively, and the resulting suspension was sonicated for 2

minutes. The acetonitrile suspension of photocatalyst and probe (100 μL) was added into an ESR tube. Under the irradiation of a 25 W blue LED light (420 < λ_{em} < 490 nm, 5.00 mW cm^{-2}) for 5 minutes, ESR measurements were carried out at room temperature in air. In the control experiments, 10.0 μL of benzylamine was added into the testing mixture.

Singlet oxygen quantum yield measurements. Before the experiment, the absorption of the photosensitizers, including PTC-1(2H) and H_2CBPP, at the excitation wavelength of 550 nm was kept as *ca*. 0.1. Measurements of singlet oxygen quantum yield were taken at 550 nm excitation in O_2-saturated solutions at room temperature with TPP ($\Phi_\Delta = 0.70$) in toluene as reference,[3] by comparing the integrated intensity of singlet oxygen phosphorescence emission around 1270 nm measured with an Edinburgh FLS 980 instrument spectrofluorimeter.

The singlet oxygen quantum yields were calculated by using equation $\Phi_\Delta^{s} = \Phi_\Delta^{r}(I_\Delta^{s} A_r n_s^2)/(I_\Delta^{r} A_s n_r^2)$, where the super/subscripts s and r refer to the sample and the reference compound, respectively. Φ_Δ is the singlet oxygen quantum yield, I_Δ is the integrated intensity of singlet oxygen phosphorescence emission, A is the absorbance at the excitation wavelength, n is the refractive index of the solvent.

Photo-driven amine oxidation. For homogeneous photocatalysis, typically, amine substrate (0.100 mmol) and photosensitizer (0.300 μmol based on porphyrin unit) were dissolved in 500 μL toluene-d_8 in a 5.0 mL glass vessel. However, for the heterogeneous photocatalysis, the starting materials including amine substrate (0.100 mmol) and solid photosensitizer (3.00 μmol based on the porphyrin unit) were suspended in 1.0 mL CD_3CN in a 5.0 mL glass vessel. Before the reaction, oxygen was bubbled into the

mixture for 5.0 min using an oxygen balloon. The reaction vessel in a water bath was then irradiated with a blue 25 W LED light ($420 < \lambda_{em} < 490$ nm, 5.00 mW cm^{-2}) for the present aerobic oxidation of amine derivatives, and the conversion was determined by ^{1}H NMR.

In the heterogeneous recycle test, the photo-driven aerobic oxidation of benzylamine (0.100 mmol) in the presence of PTC-1(2H) (1.00 μmol) was carried out. After each cycle of reaction, another benzylamine (0.100 mmol) was added into the resulting mixture for the next cycle of reaction.

Theoretical simulations. Before the preparation of PTC-1(2H), its formation energy was calculated by density functional theory (DFT). The structure of each compounds was firstly optimized on the basis of B3LYP-D3(BJ)/6-31G(D).[4-6] The high level single point energy of each structure was calculated on the level of B3LYP-D3(BJ)/6-311G(2D,2P),[7] Supplementary Data 1. The formation energy E_f of PTC-1(2H) is calculated according to the reported literature procedures,[8] shown by Eq. **I**:

$E_f = [(E_{cage} + xE_{water}) - (mE_{aldehyde} + nE_{amine})] / xn$ (**I**)

where E_{cage} is the energy of the cage formed, E_{water} is the energy of the water produced in the reaction, $E_{aldehyde}$ is the energy of the aldehyde, and E_{amine} is the energy of the imine. The number of aldehyde reactants is m, n is the number of amine precursors, and xn is the number of imine bonds formed, equivalent to the number of water molecule produced in the reaction. All calculations are carried out by Gaussian 09. D.01 software package.[9]

Furthermore, we employed density functional theory (DFT) to calculate the frontier molecular orbital distribution of the PTC-1(2H) on the basis of B3LYP/6-311G(D),[4-7] using Gaussian 09 D.01 software package.[9] It can be seen from Supplementary Fig. 18

that the HOMO and LUMO of the compound are uniformly distributed on the three porphyrin units, indicating the lack of obvious orbital interaction between the three porphyrin molecules after the formation of the cage.

All structures were optimized on the basis of B3LYP-D3BJ/6-311G(D)[4-7] using Gaussian 09.D 01 version software package.[9]

Supplementary Figures and Supplementary Tables

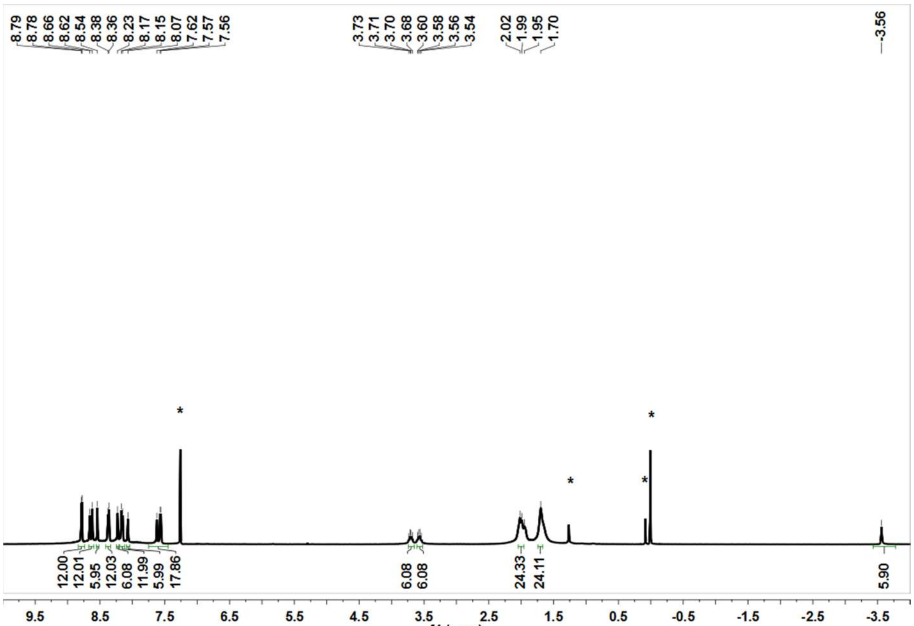

Supplementary Fig. 1 ^{1}H NMR spectrum of (*R*)-PTC-1(2H) (* denotes $CDCl_3$ solvent impurity).

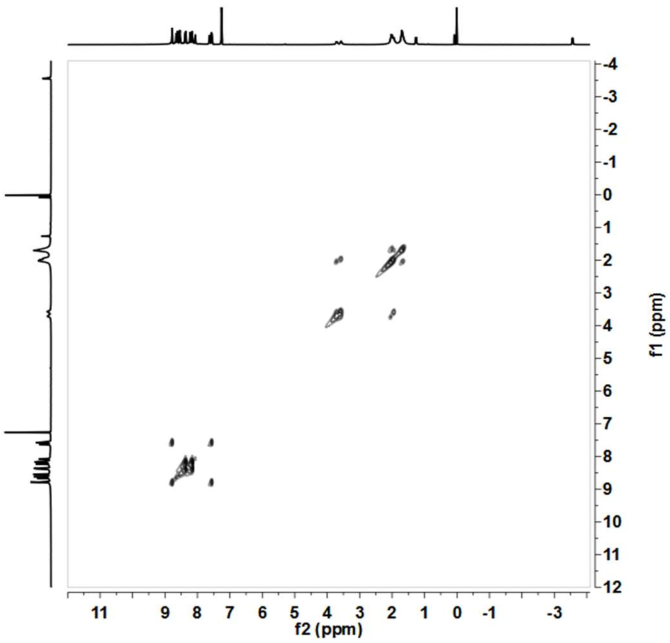

Supplementary Fig. 2 ^{1}H-^{1}H COSY spectrum of (*R*)-PTC-1(2H).

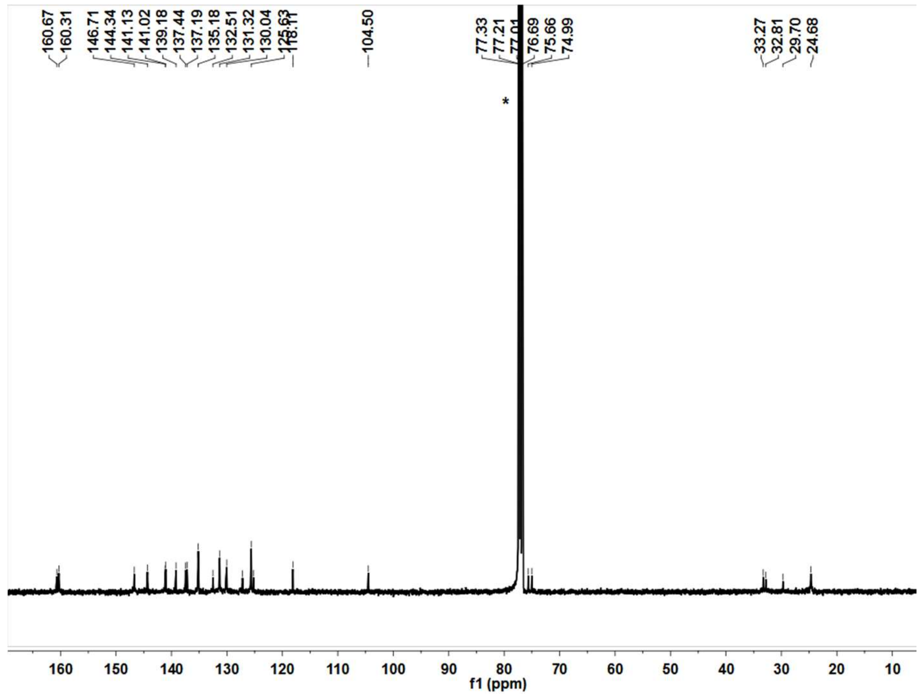

Supplementary Fig. 3 ^{13}C NMR spectrum of (*R*)-PTC-1(2H) (* denotes $CDCl_3$ solvent impurity).

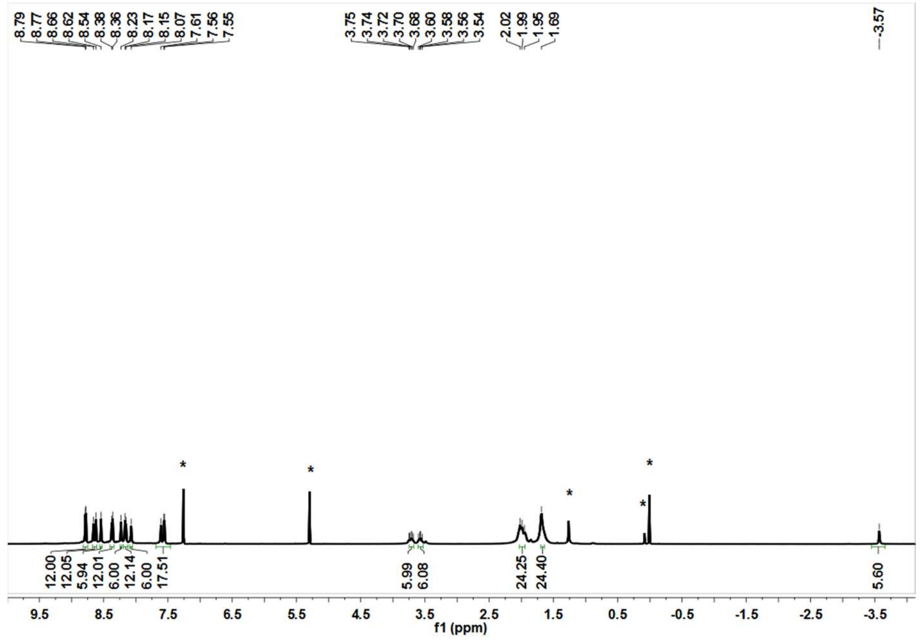

Supplementary Fig. 4 ^{1}H NMR spectrum of (*S*)-PTC-1(2H) (* denotes $CDCl_3$ solvent impurity and CH_2Cl_2 inside cage).

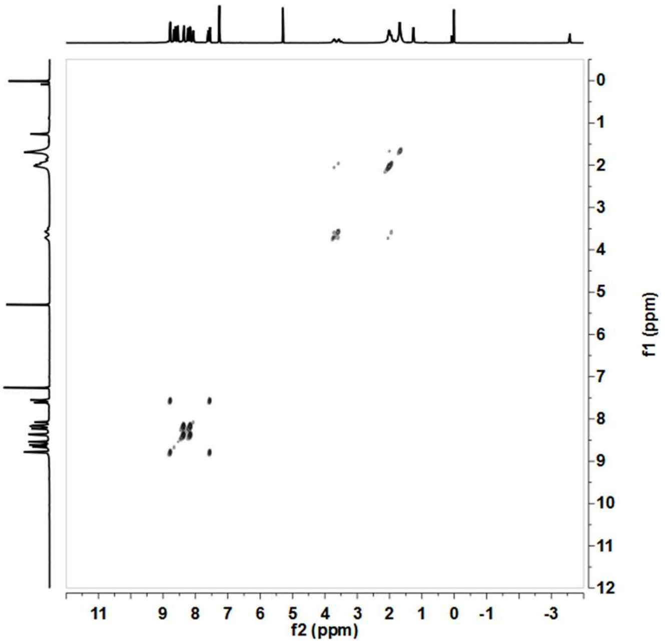

Supplementary Fig. 5 ^1H-^1H COSY spectrum of (*S*)-PTC-1(2H).

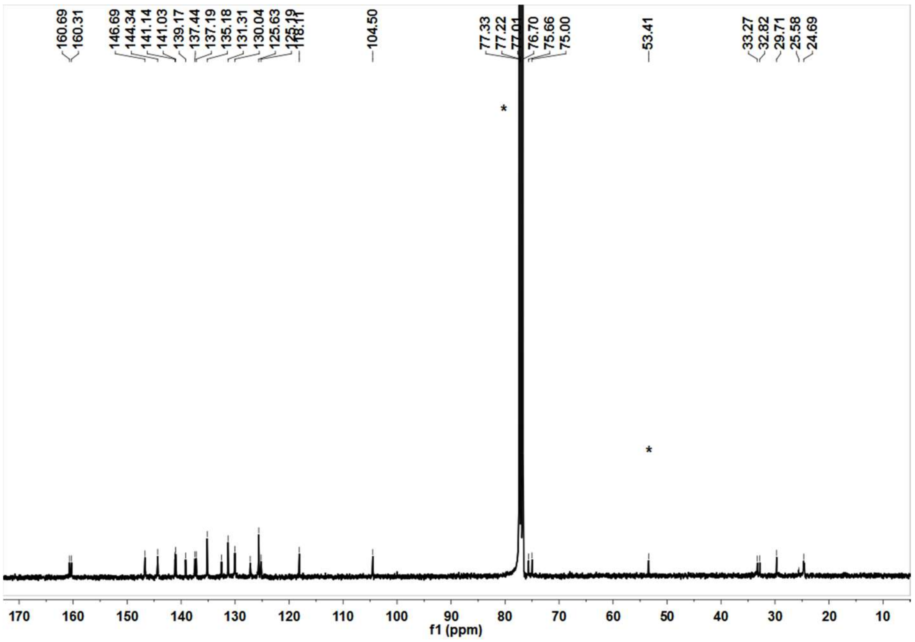

Supplementary Fig. 6 ^{13}C NMR spectrum of (*S*)-PTC-1(2H) (* denotes $CDCl_3$ solvent impurity and CH_2Cl_2).

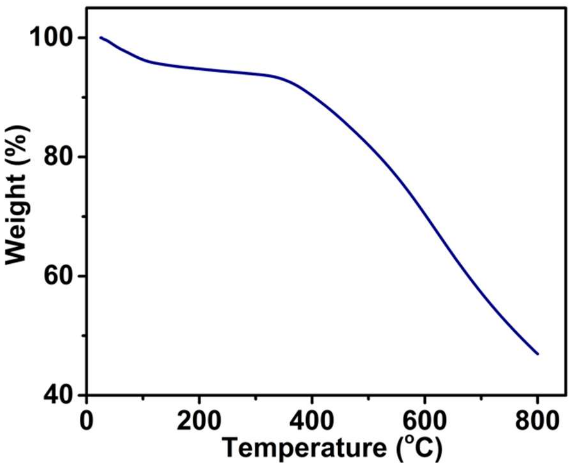

Supplementary Fig. 7 TGA curve of PTC-1(2H) in the range of 25-800°C under N_2 atmosphere. Source data are provided as a Source Data file.

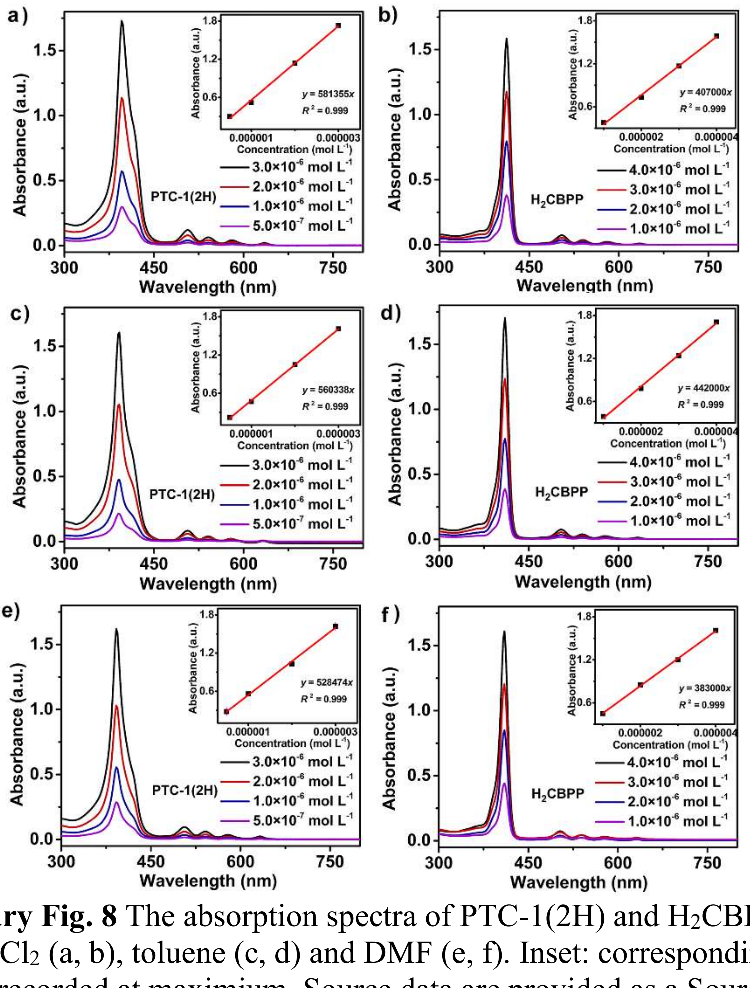

Supplementary Fig. 8 The absorption spectra of PTC-1(2H) and H_2CBPP in different solvents. CH_2Cl_2 (a, b), toluene (c, d) and DMF (e, f). Inset: corresponding Beer-Lambert plot recorded at maximium. Source data are provided as a Source Data file.

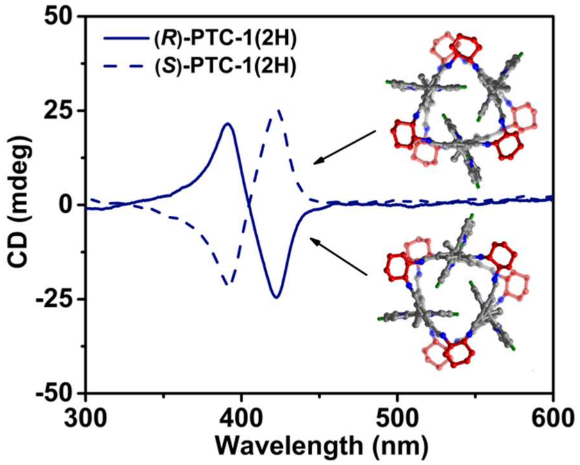

Supplementary Fig. 9 CD spectra of (*R*)/(*S*)-PTC-1(2H) in CH_2Cl_2 with a concentration of 1.0 ×10^{-6} mol L^{-1}. Source data are provided as a Source Data file.

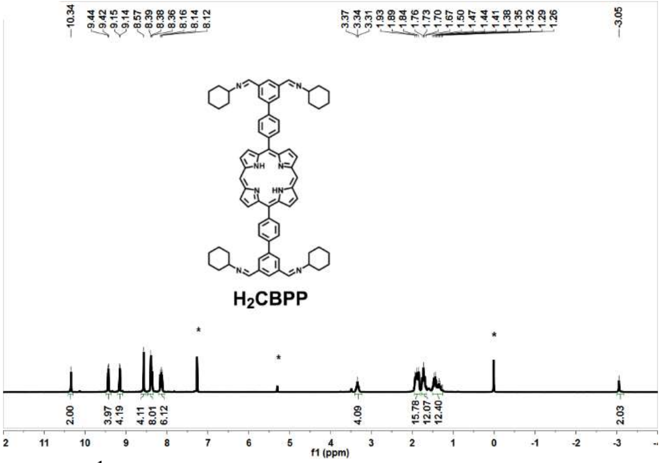

Supplementary Fig. 10 ^{1}H NMR spectrum of H_2CBPP (* denotes $CDCl_3$ solvent impurity and CH_2Cl_2 included in sample).

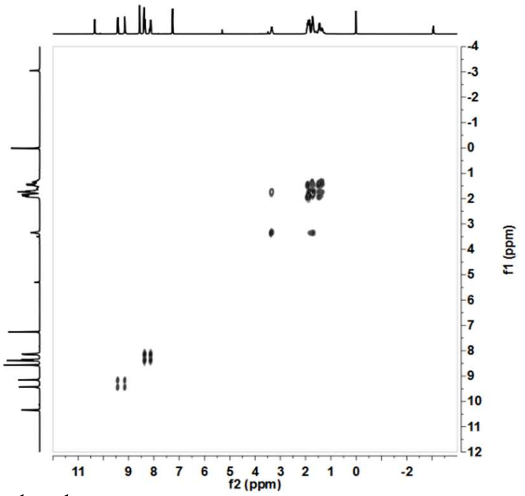

Supplementary Fig. 11 ^{1}H-^{1}H COSY spectrum of H_2CBPP.

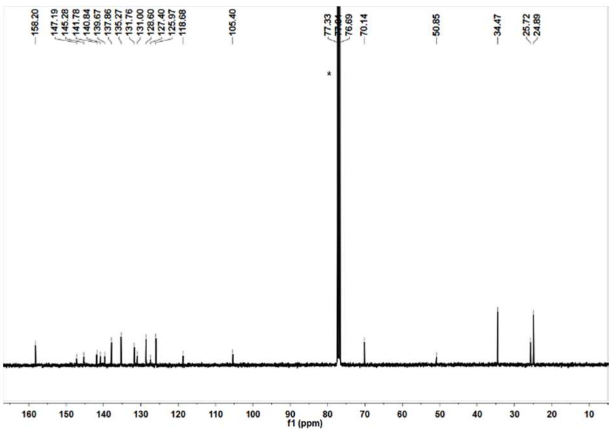

Supplementary Fig. 12 ^{13}C NMR spectrum of H_2CBPP (* denotes $CDCl_3$ solvent impurity and CH_2Cl_2 included in sample).

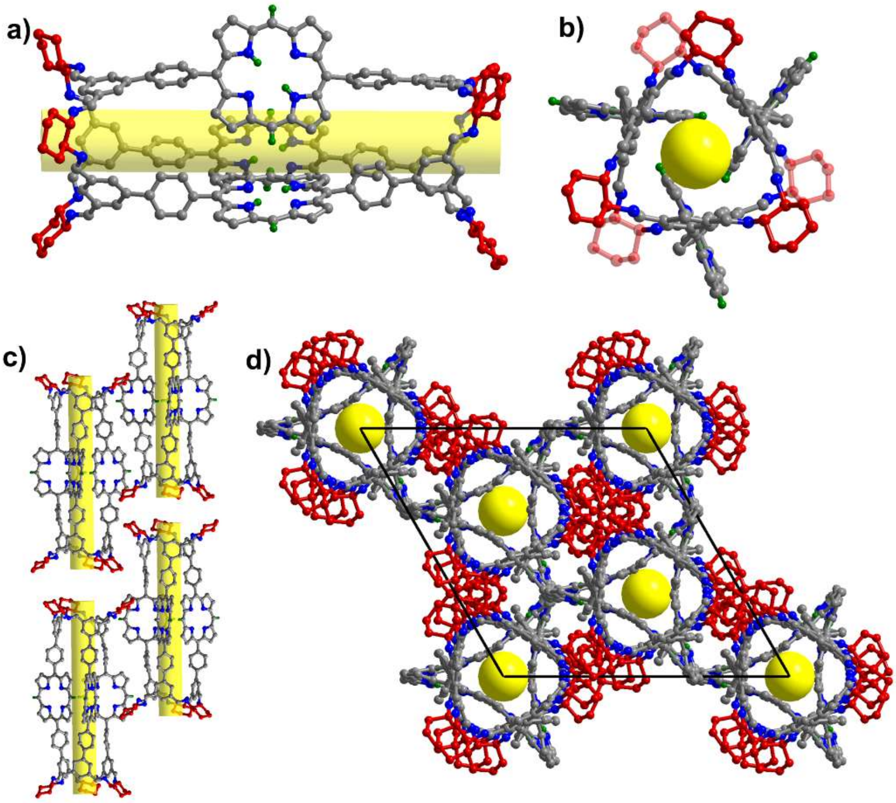

Supplementary Fig. 13 Single crystal structure of molecular organic cage (*S*)-PTC-1(2H). **a** side view and **b** top view; **c** window-to-window stacking mode of neighboring molecular organic cages; **d** packing profile along the direction of [001] (porphyrin C, grey; cyclohexanediamine C, red; N, blue; H, green; yellow tubes and balls represent the open one-dimenrsional channel and window, respectively; all selected hydrogen atoms omitted for clarity).

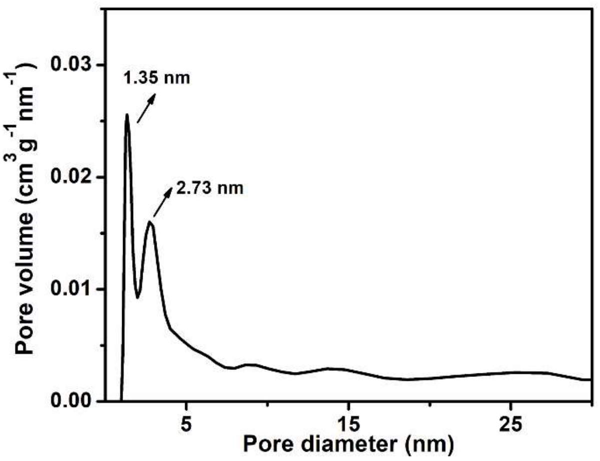

Supplementary Fig. 14 DFT pore size distribution of PTC-1(2H) based on the N_2 adsorption isotherm. Source data are provided as a Source Data file.

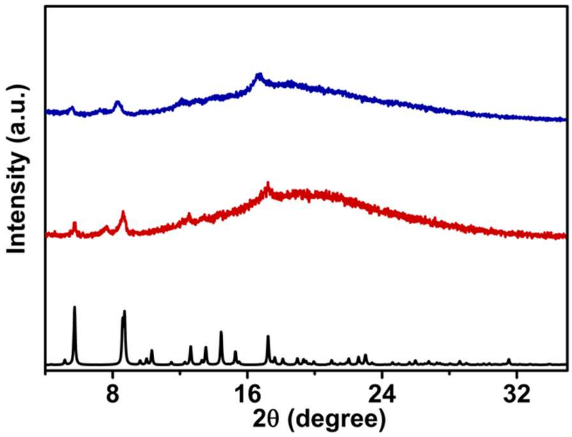

Supplementary Fig. 15 Powder X-ray diffraction profiles for as-prepared PTC-1(2H) (blue) and degassed PTC-1(2H) (red) in comparison with a simulated powder pattern (black) based on the PTC-1(2H) single-crystal structure without considering the solvent molecules. Source data are provided as a Source Data file.

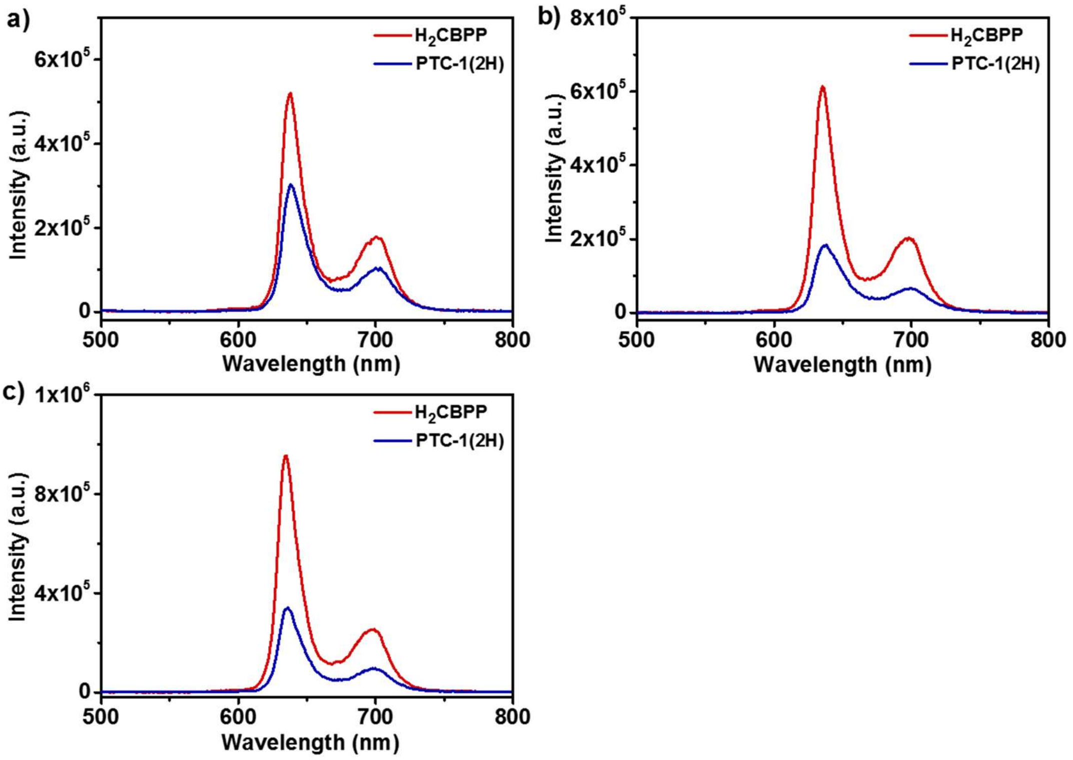

Supplementary Fig. 16 Fluorescence spectra of PTC-1(2H) and H_2CBPP in different solvents. CH_2Cl_2 (a), toluene (b) and DMF (c) (3.0×10^{-6} mol L^{-1} for the porphyrin unit). Source data are provided as a Source Data file.

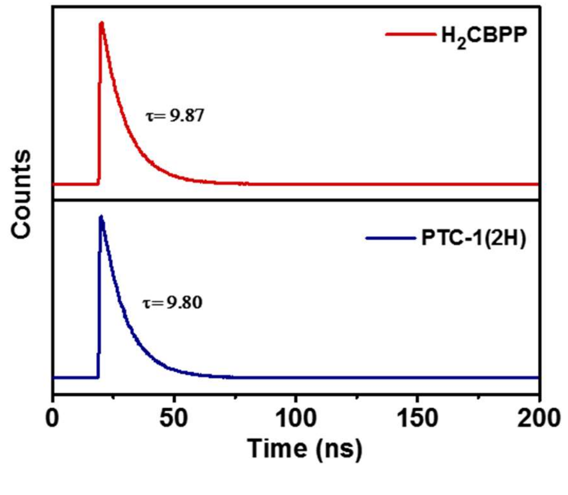

Supplementary Fig. 17 Fluorescence decay curves of PTC-1(2H) and H_2CBPP in toluene with a concentration of 3.0×10^{-6} mol L^{-1} for the porphyrin unit. The time profiles of fluorescence decays were obtained with excitation at 405 nm by an EPL laser. Source data are provided as a Source Data file.

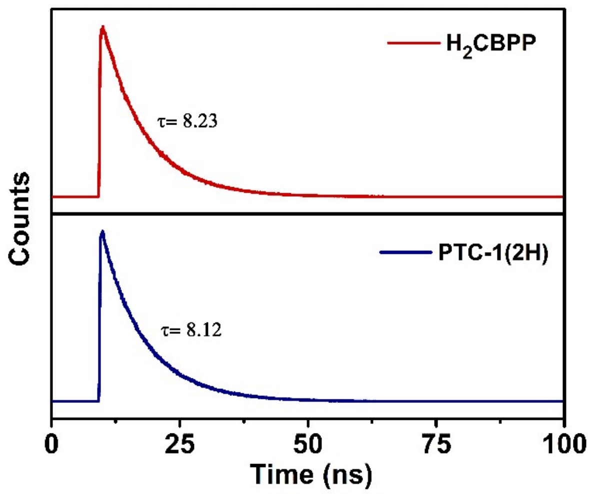

Supplementary Fig. 18 Fluorescence decay curves of PTC-1(2H) and H_2CBPP in CH_2Cl_2 with a concentration of 3.0 $\times 10^{-6}$ mol L^{-1} for the porphyrin unit. The time profiles of fluorescence decays were obtained with excitation at 405 nm by an EPL laser. Source data are provided as a Source Data file.

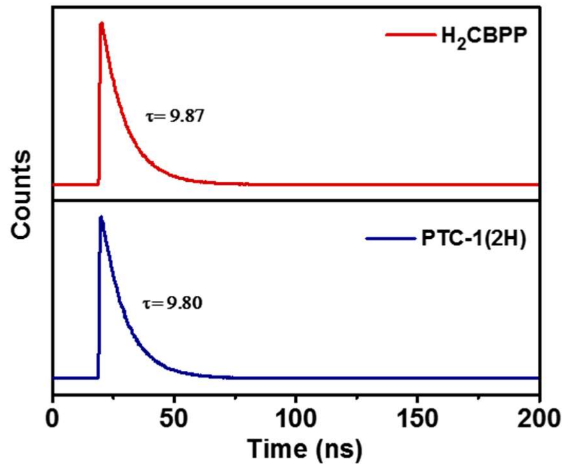

Supplementary Fig. 19 Fluorescence decay curves of PTC-1(2H) and H_2CBPP in DMF with a concentration of 3.0 $\times 10^{-6}$ mol L^{-1} for the porphyrin unit. The time profiles of fluorescence decays were obtained with excitation at 405 nm by an EPL laser. Source data are provided as a Source Data file.

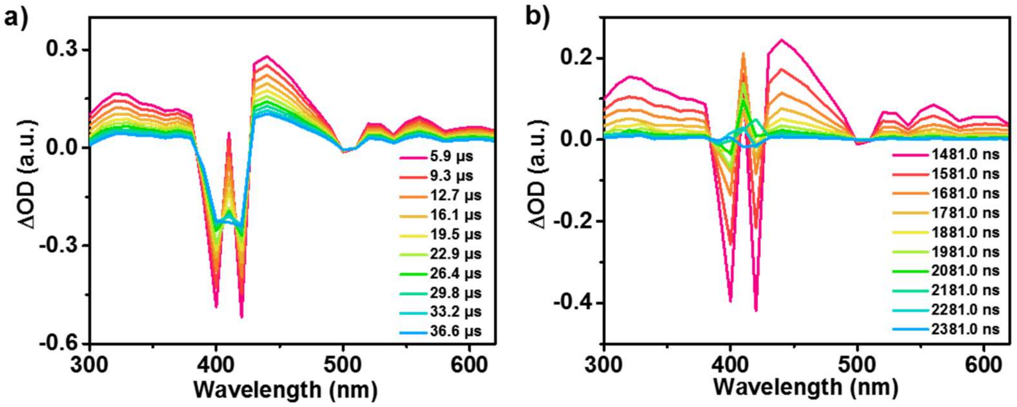

Supplementary Fig. 20 Nanosecond TA spectra of H_2CBPP after 355 nm excitation in N_2 (a) and air (b). Source data are provided as a Source Data file.

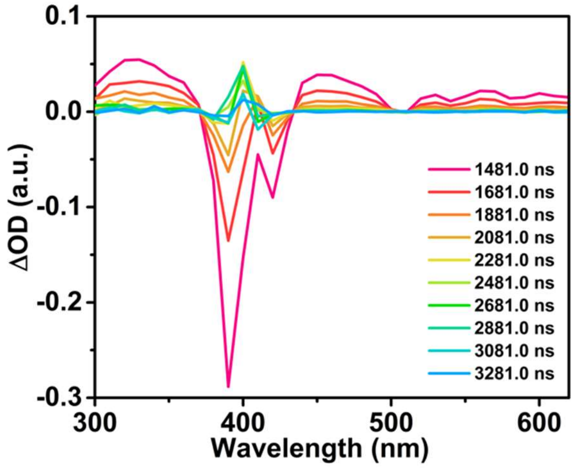

Supplementary Fig. 21 Nanosecond TA spectra of PTC-1(2H) after 355 nm excitation in air. Source data are provided as a Source Data file.

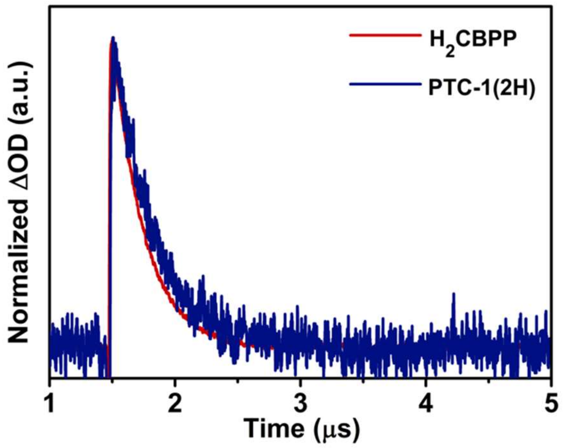

Supplementary Fig. 22 Normalized kinetics at 450 nm in nanosecond TA spectra of, PTC-1(2H) and H_2CBPP after 355 nm excitation in air. Source data are provided as a Source Data file.

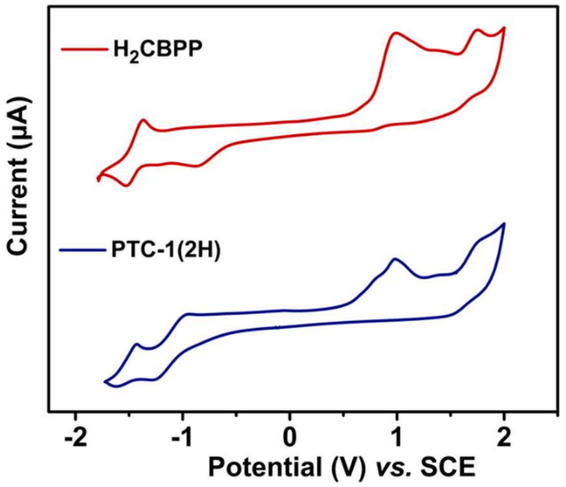

Supplementary Fig. 23 Cyclic voltammograms of PTC-1(2H) and H_2CBPP in CH_2Cl_2.

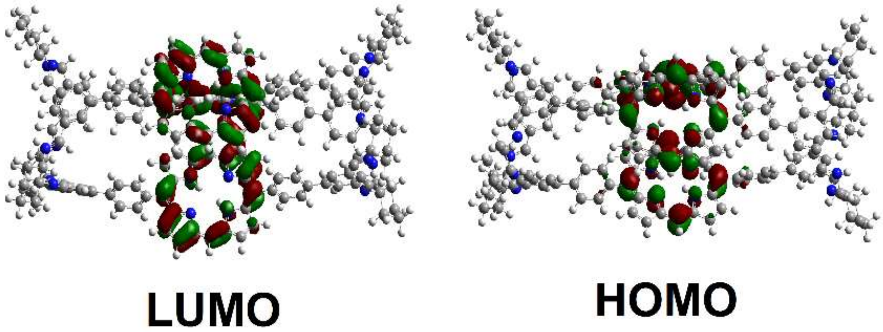

Supplementary Fig. 24 Diagram of frontier molecular orbitals (LUMO and HOMO) of PTC-1(2H).

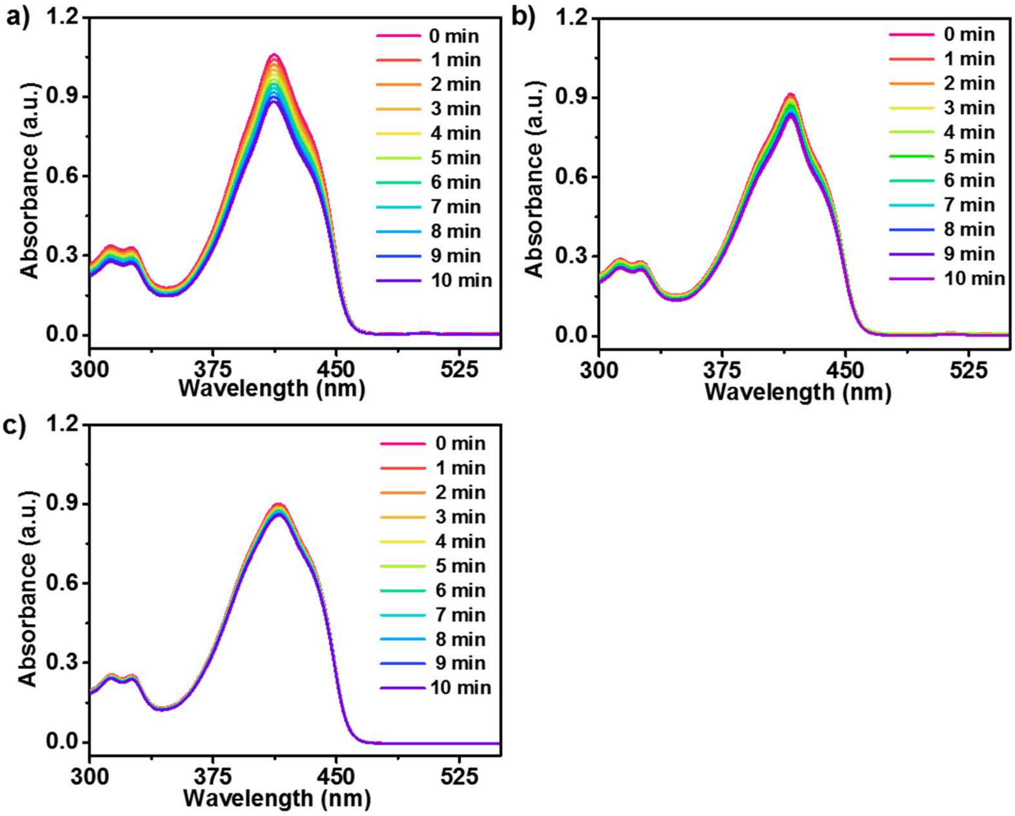

Supplementary Fig. 25 Photo-bleach experiments based on the oxidation of DPBF in DMF. Time-dependent absorption spectra of DPBF upon irradiation at $\lambda > 510$ nm with the presence of homogeneous catalysts H_2CBPP (a), TPP (b), and blank (c) in air. Source data are provided as a Source Data file.

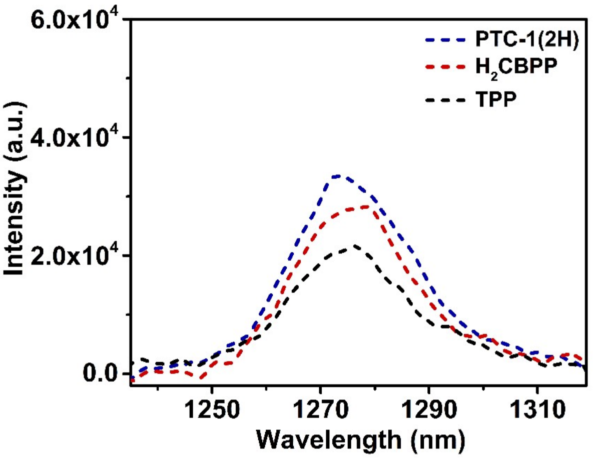

Supplementary Fig. 26 Singlet oxygen phosphorescence spectra of PTC-1(2H), H_2CBPP, and TPP excited at 550 nm in toluene.

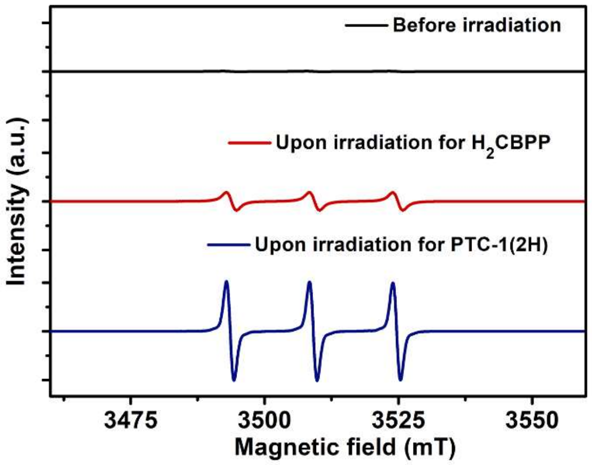

Supplementary Fig. 27 ESR detection of $^1O_2$ generation over PTC-1(2H) *vs*. H_2CBPP trapped by TEMP in toluene. Source data are provided as a Source Data file.

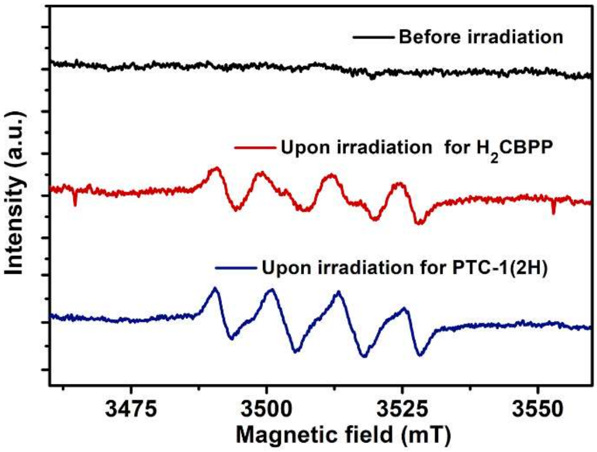

Supplementary Fig. 28 ESR detection of $O_2^{\bullet-}$ generation over PTC-1(2H) *vs*. H_2CBPP trapped by DMPO in toluene. Source data are provided as a Source Data file.

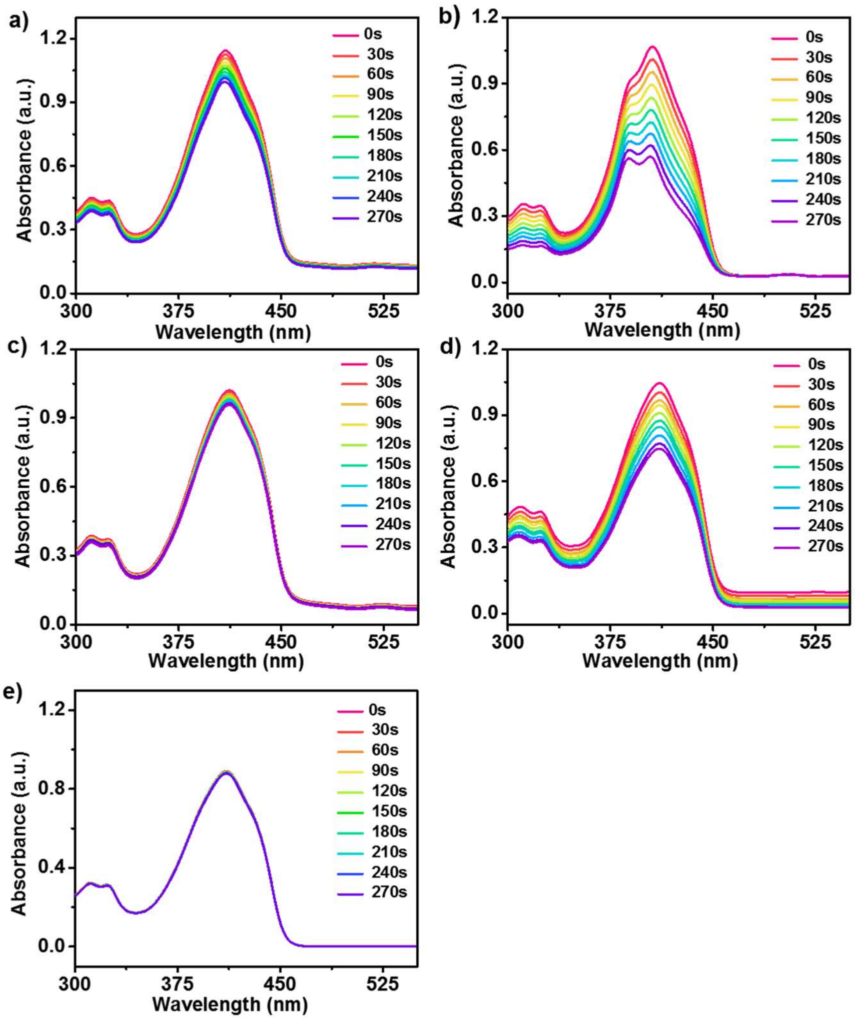

Supplementary Fig. 29 Photo-bleach experiments based on the oxidation of DPBF upon various photocatalyst in CH_3CN. Time-dependent absorption spectra of DPBF upon irradiation of $\lambda > 510$ nm in the presence of heterogeneous catalysts H_2CBPP (a), PTC-1(2H) (b), PCN-224 (c), PCN-222 (d), and blank (e) in air. Source data are provided as a Source Data file.

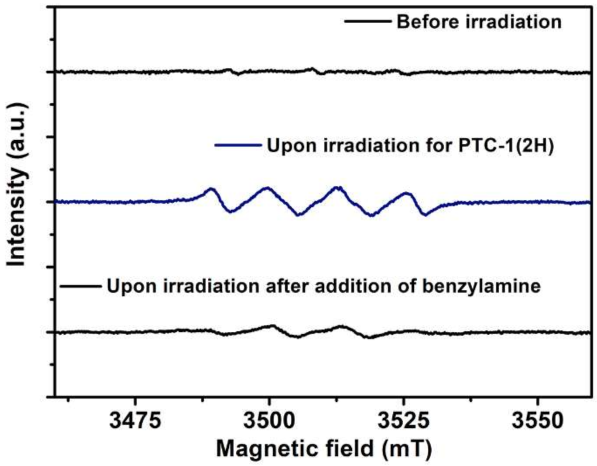

Supplementary Fig. 30 ESR detection of $O_2^{\bullet-}$ generation over PTC-1(2H) trapped by DMPO in CH_3CN.

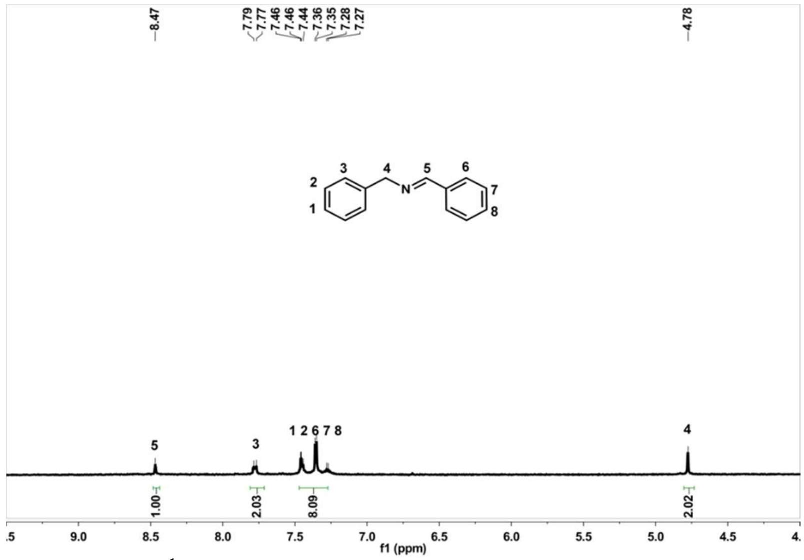

Supplementary Fig. 31 ^{1}H NMR spectrum of N-benzylidenebenzylamine inside the pores of PTC-1(2H) framework after catalysis.

Supplementary Table 1 Mass spectroscopic and elemental analysis data for H_2CBPP and (*R*)/(*S*)-PTC-1(2H).[a]

Compound	m/z[b]	Analysis		
		C	H	N
H_2CBPP	1051.01 (1051.61)	82.35 (82.25)	7.21 (7.09)	10.62 (10.66)
(*R*)-PTC-1(2H)	2649.53 (2649.25)	76.96 (77.07)[c]	5.88 (5.40)[c]	11.66 (11.89)[c]
(*S*)-PTC-1(2H)	2649.34 (2649.25)	81.22 (81.60)	5.54 (5.70)	12.87 (12.69)

[a] Calculated values given in parentheses. [b] By MALDI-TOF mass spectrometry. [c] Contain 1.5 equiv. solvated $CHCl_3$.

Supplementary Table 2 Crystal data and structure refinements for PTC-1(2H).

Compound	(*R*)-PTC-1(2H)	(*S*)-PTC-1(2H)
formula	$C_{180}H_{150}N_{24}$	$C_{180}H_{150}N_{24}$
fw	2649.23	2649.23
crystal system	trigonal	trigonal
space group	*R*32	*R*32
a /Å	22.7028(2)	22.6926(6)
b /Å	22.7028(2)	22.6926(6)
c /Å	67.7954(12)	67.010(2)
α /°	90	90
β /°	90	90
γ /°	120	120
V /Å^3	30261.4(8)	29884.1(19)
Z	6	6
θ range (deg)	1.955-68.244	3.4610-21.134
Density (g/cm^3)	0.872	0.883
μ(mm^{-1})	0.405	0.053
F(000)	8388	8388
R_1 (I>2θ)[a]	0.0889	0.1232
R_{w2} for all[b]	0.2628	0.3504
GOF on F^2	1.074	1.057
CCDC No.	1913971	1913972

[a] $R_1 = \Sigma|F_o-|F_c||/\Sigma|F_o|$. [b] $wR_2 = [\Sigma w(F_o^2-F_c^2)^2/\Sigma w(F_o^2)^2]^{1/2}$

Supplementary Table 3 Photophysical and electrochemical data for H_2CBPP and PTC-1(2H) in CH_2Cl_2 and DMF.

Compound	UV/vis spectra	fluorescence spectra			
	λ_{max} (nm)	λ_{ex} (nm)	λ_{em} (nm)	τ (ns)	φ (%)
PTC-1(2H)[a]	390, 506, 541, 578, 632	405	637, 700	8.12	1.34
H_2CBPP[a]	411, 504, 540, 577, 630	405	636, 698	8.23	1.50
PTC-1(2H)[b]	392, 506, 541, 579, 632	405	636, 699	10.30	2.34
H_2CBPP[b]	410, 505, 540, 577, 631	405	635, 698	11.41	2.38

[a] In CH_2Cl_2; [b] in DMF.

Supplementary References

1. Senge, M. O. *et al.* Synthesis of meso-substituted ABCD-type porphyrins by functionalization reactions. *Eur. J. Org. Chem.* **2010**, 237-258, (2010).

2. Sheldrick, G. Crystal structure refinement with SHELXL. *Acta Cryst C* **71**, 3-8, (2015).

3. Shao, W. *et al.* Photophysical properties and singlet oxygen generation of three sets of halogenated corroles. *J. Phys. Chem. B* **116**, 14228-14234, (2012).

4. Becke, A. D. Density-functional thermochemistry. III. The role of exact exchange. *J. Chem. Phys.* **98**, 5648-5652, (1993).

5. Grimme, S., Ehrlich, S. & Goerigk, L. Effect of the damping function in dispersion corrected density functional theory. *J. Comp. Chem.* **32**, 1456-1465, (2011).

6. Hariharan, P. C. & Pople, J. A. Influence of polarization functions on MO hydrogenation energies. *Theor. Chim. Acta.* **28**, 213-222, (1973).

7. Krishnan, R., Binkley, J. S., Seeger, R. & Pople, J. A. Self-consistent molecular orbital methods. XX. A basis set for correlated wave functions. *J. Chem. Phys.* **72**, 650-654, (1980).

8. Santolini, V., Miklitz, M., Berardo, E. & Jelfs, K. E. Topological landscapes of porous organic cages. *Nanoscale* **9**, 5280-5298, (2017).

9. M. J. Frisch, et al., Gaussian 09, Revision D.01; Gaussian, Inc.: Wallingford CT, (2010).
