# Supplementary material for: Elucidating heterogeneous photocatalytic superiority of microporous porphyrin organic cage
Source: Nat Commun. 2020 Feb 26;11:1047. doi: 10.1038/s41467-020-14831-x (PMC7044162; doi:10.1038/s41467-020-14831-x)
Supplement: Supplementary file 2 — Description of Additional Supplementary Files [file 41467_2020_14831_MOESM2_ESM.pdf]

Description of Additional Supplementary Files

File Name: Supplementary Data 1
Description: Cartesian coordinates (Å), SCF energies, and free
energies at 298.15 K and 1 atm for the optimized structures. All energies are given in Hartree and on the basis of B3LYP-D3(BJ)/6-31G(D)//6-311G(2D,2P)
